# A versatile insulin analog with high potency for both insulin and insulin-like growth factor 1 receptors: Structural implications for receptor binding

**DOI:** 10.1074/jbc.RA118.004852

**Published:** 2018-09-13

**Authors:** Martina Chrudinová, Lenka Žáková, Aleš Marek, Ondřej Socha, Miloš Buděšínský, Martin Hubálek, Jan Pícha, Kateřina Macháčková, Jiří Jiráček, Irena Selicharová

**Affiliations:** From the Institute of Organic Chemistry and Biochemistry, Czech Academy of Sciences, Flemingovo n. 2, 166 10 Prague 6, Czech Republic

**Keywords:** insulin, insulin-like growth factor (IGF), structure-function, insulin receptor, protein design, kinetics, binding, Site 1

## Abstract

Insulin and insulin-like growth factor 1 (IGF-1) are closely related hormones involved in the regulation of metabolism and growth. They elicit their functions through activation of tyrosine kinase–type receptors: insulin receptors (IR-A and IR-B) and IGF-1 receptor (IGF-1R). Despite similarity in primary and three-dimensional structures, insulin and IGF-1 bind the noncognate receptor with substantially reduced affinity. We prepared [d-His^B24^, Gly^B31^, Tyr^B32^]-insulin, which binds all three receptors with high affinity (251 or 338% binding affinity to IR-A respectively to IR-B relative to insulin and 12.4% binding affinity to IGF-1R relative to IGF-1). We prepared other modified insulins with the aim of explaining the versatility of [d-His^B24^, Gly^B31^, Tyr^B32^]-insulin. Through structural, activity, and kinetic studies of these insulin analogs, we concluded that the ability of [d-His^B24^, Gly^B31^, Tyr^B32^]-insulin to stimulate all three receptors is provided by structural changes caused by a reversed chirality at the B24 combined with the extension of the C terminus of the B chain by two extra residues. We assume that the structural changes allow the directing of the B chain C terminus to some extra interactions with the receptors. These unusual interactions lead to a decrease of dissociation rate from the IR and conversely enable easier association with IGF-1R. All of the structural changes were made at the hormones' Site 1, which is thought to interact with the Site 1 of the receptors. The results of the study suggest that merely modifications of Site 1 of the hormone are sufficient to change the receptor specificity of insulin.

## Introduction

**Figure 1.**
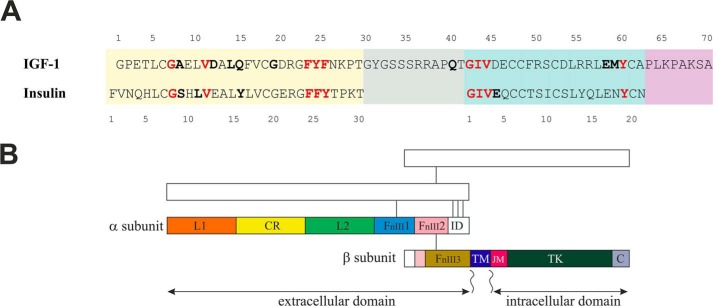
**Amino acid sequences of human insulin and IGF-1 (*A*) and schematic organization of IR and IGF-1R domains (*B*).** *A*, insulin B chain and corresponding B domain in IGF-1 are highlighted by a *yellow background*. The *green background* indicates A chain and A domain. IGF-1 C and D domains are shown in *gray* or in *violet*. Amino acid residues depicted in *boldface type* were shown to contact the cognate receptor Site 1 in the crystal structures (6, 11–14). *Red*, residues found to interact with the receptors in the same fashion. *B*, structural domains of the receptor (αβ) dimer are marked on one half of the dimer, the second part is *sketched*. Subunits are disulfide-linked, as indicated by *connecting lines*. Domains are named as follows: leucine-rich (*L*), cysteine-rich (*CR*), fibronectin type III (*FnIII*), insert (*ID*), transmembrane (*TM*), juxtamembrane (*JM*), tyrosine kinase (*TK*), and C-terminal (*C*). For a review, see Belfiore *et al.* (2, 3)

Insulin and insulin-like growth factors 1 and 2 (IGF-1 and IGF-2)^3^ are closely related protein hormones. Insulin is a key modulator of metabolism, whereas IGFs are factors indispensable for growth and development (1). They elicit their functions through activation of tyrosine kinase–type receptors (insulin receptor isoforms A and B (IR-A and IR-B), IGF-1 receptor (IGF-1R), or their hybrid forms) in cytoplasmic membranes of cells (2, 3). Malfunction of these hormones' complex signaling systems leads to both types of diabetes mellitus, increased cancer risk, and other life-threatening disorders (3). Amino acid sequences of insulin and IGF-1 and schematic organization of IR and IGF-1R domains are shown in (Fig. 1). Insulin is a two-chain molecule, where A and B chains are connected by two cysteine bridges, and the third disulfide bridge is in the A chain. IGF-1 is formed by a single chain organized into A, B, C, and D domains, where A and B domains are highly homologous to the A and B chains of insulin and share the same three-dimensional structure. The C terminus of the B domain in IGF-1 is connected to the N terminus of the A domain by a C domain. The D domain extends the C terminus of the A domain.

The receptors are members of the receptor tyrosine kinase family. They are disulfide-linked (αβ)_2_ homodimers. Ligand binding to the receptor exhibits complex kinetics, characterized by a curvilinear Scatchard plot and negative cooperativity (4, 5). The commonly accepted assumption is that two distinct binding sites (Site 1 and Site 2) on a ligand interact with two receptor sites (Site 1 and Site 2, respectively) located on separate α subunits to create a high-affinity binding complex that is necessary for activation of the tyrosine kinase. The binding is asymmetrical; thus, only one ligand is bound per homodimer in the high-affinity complex (5–7).

Despite similarity in their primary and three-dimensional structures, insulin and IGF-1 bind the noncognate receptor with substantially reduced affinity. The structural basis for this discrimination is as yet unclear (6, 8). Insulin and IGF-1 receptors have a similar binding Site 1 that can accommodate both hormones. The difference in affinity of insulin and IGF-1 for the receptors results from different residues interacting with specificity-conferring regions on the two receptors. The studies of chimeric insulin/IGF-1 receptors ascribed insulin specificity mainly to the N-terminal sequence 1–68 of the leucine-rich (L1) domain of IR and IGF-1 specificity to the sequence 191–290 of the cysteine-rich (CR) domain of the IGF-1R (Fig. 1) (9, 10).

Major progress in the understanding of insulin and IGF-1 binding to their receptors was achieved through the recent solving of structures of a few ligand–receptor complexes that were engineered for protein crystallography (6, 11–14). These structures provided a wealth of information about the receptor's Site 1 interactions. However, the exact nature of interactions during Site 2 binding and the structural changes of the receptor leading to the tyrosine kinase activation are still not completely understood. Conformational change required for bridging of the receptor's Sites 1 and 2 is supposed to initiate signal transmission to the kinase region. Constraints holding transmembrane regions apart are thus released, and receptor kinase autophosphorylation is triggered (11, 15). The extent of conformational change was recently visualized using single-particle cryo-EM of full-length human IR reconstituted into lipid nanodiscs (16). The location of Site 2 in insulin and the amino acids important for the interaction were deduced from mutagenesis studies. Site 2 on the receptor was mapped near the junction of fibronectin type III domains 1 and 2 (FnIII1-FnIII2) of the α subunit (17). Recently, a structure of IR ectodomain with bound insulin, obtained using single-particle cryo-EM, was published (18). It positioned the Site 2 location within the first fibronectin-like domain (FnIII1). Rather different and much more restricted Site 2 interacting residues in insulin sequence (virtually Cys^A7^ and Thr^A8^ only) were detected in the cryo-EM analysis, compared with the mutagenesis studies (Thr^A8^, Ile^A10^, Ser^A12^, Leu^A13^, Glu^A17^, His^B10^, Glu^B13^, and Leu^B17^) (8, 19). Moreover, the IR–ectodomain dimer identified by cryo-EM did not resemble the crystallographic symmetry–generated dimer. Issues arising from these findings will need further clarification.

Concerning the IGF-1 binding, it is not clear to what extent Site 2 is important (19). It remains possible that the receptor's Site 2 is located in different parts of IGF-1R compared with IR (12). It was also proposed that Site 1 of IGF-1 is extended to the IGF-1 C domain and interacts also with the CR domain of IGF-1R (20). Unfortunately, the last three residues at the C termini of the B chain/domain of insulin and IGF-1 and the C and D domains of IGF-1 were not traceable in any of the structures of hormone–receptor complexes solved so far.

A number of studies have supported a critical role of insulin residues B24 and B25 in receptor binding (8). On the other hand, residues B26–B30 are not required for IR binding. Des-(B26–B30)-pentapeptide-B25-carboxamide insulin had full potency (21). In the past, we prepared a series of des-(B27-B30)-tetrapeptide-B26-carboxamide insulin (-DTI-NH_2_) analogs with a modified B26 position that have severalfold increased binding to the IR (22, 23). However, the residues B26–B30 of insulin are crucial for the formation of insulin dimers, conferring thermodynamic stability and self-assembly of insulin (24, 25), and Tyr^B26^ was proposed as playing a role in the negative cooperativity of insulin (4).

Conversely, Slieker *et al.* (26) reported sensitivity of IGF-1R to structural changes in the C-terminal portion of the B chain of insulin. They prepared a series of insulin analogs, modified at B28-B29 positions that were approximately equipotent to insulin in binding to the IR but showed varying affinity to the IGF-1R. Basic amino acid residues increased, whereas acidic residues reduced relative affinity to the IGF-1R. Multiple basic residues in IGF-1 D and C domains were suggested as modulating IGF-1R specificity (27) and interacting with the CR region of IGF-1R that has negative surface electrostatic potential (28).

An insulin analog with three modifications in the C-terminal part of the B chain and with exceptionally increased (about 150-fold) affinity for IGF-1R is described in this paper. At the same time, the analog is also markedly more potent than human insulin in binding to both IR isoforms. We have designed a series of new analogs and performed several kinetic and biochemical experiments to explain this observation.

## Results

### Analog design

**Figure 2.**
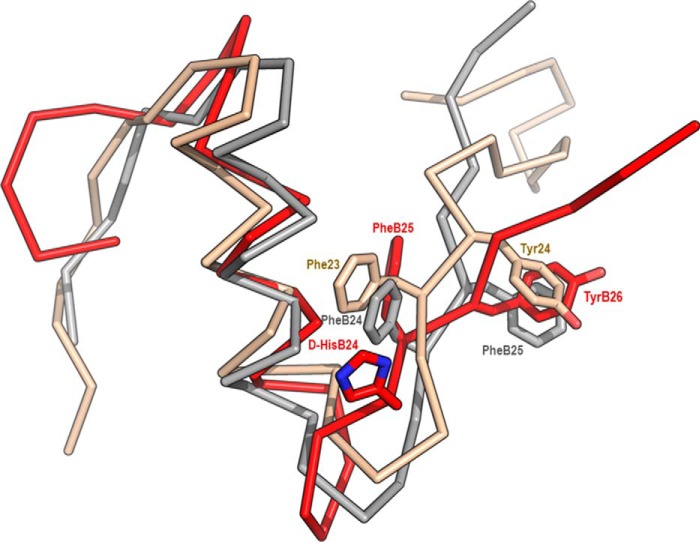
**An overlay of the B chains of human insulin with human IGF-1 and [d-His^B24^]-insulin.** Insulin (PDB code 1MSO, crystal structure) is shown in *gray*, IGF-1 (PDB code 1GZR, crystal structure) is *ocher*, and [d-His^B24^]-insulin (PDB code 2M2P, NMR structure with the lowest energy at pH 8) is *red*. Positions of downshifted d-His^B24^, Phe^B25^, and Tyr^B26^ in [d-His^B24^]-insulin are shown together with corresponding residues in insulin (Phe^B24^ and Phe^B25^) and IGF-1 (Phe^23^ and Tyr^24^).

**Figure 3.**
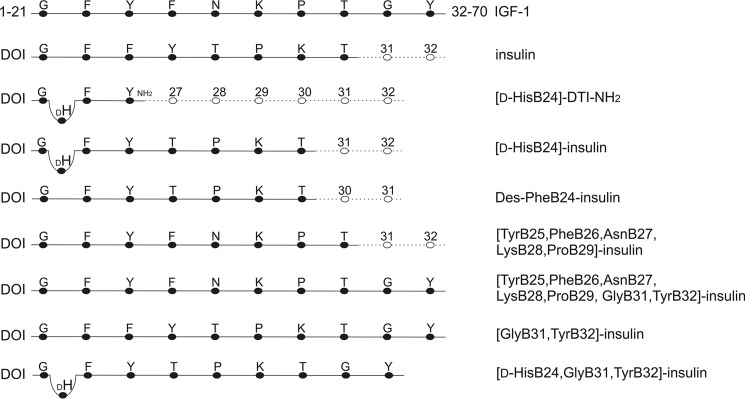
**Schematic illustration of insulin analogs and comparison of the sequences with human insulin and IGF-1.** *Single-letter codes* of amino acids are used. Vacant residues in insulin B chain are shown as *empty circles*; *numbers* represent their positions in the insulin B chain sequence. The respective sequence of human IGF-1 and its location in the amino acid chain is shown. Expected downshift of the B25 residue (and residues upward) due to the presence of d-His at B24 (*DH*) is indicated by *bending* of the *connecting line. DOI*, des-(B23-B30)-octapeptide-insulin. -*DTI-NH_2_*, des-(B27-B30)-tetrapeptide-B26-carboxamide insulin.

Previously synthetized analog [d-His^B24^]-insulin (29) binds both IR-A and IR-B with higher affinity than human insulin (Table 1). NMR structure was also previously determined (29). The main structural feature of this analog was caused by the reverse chirality of the B24Cα atom that swayed the d-His^B24^ side chain into the solvent. The pocket vacated by Phe^B24^ was filled by Phe^B25^, which mimicked the Phe^B24^ side and main chains. The Phe^B25^ downshift to the Phe^B24^ position resulted in a subsequent downshift of Tyr^B26^ into the B25 site and the departure of B26–B30 residues away from the insulin core (Fig. 2). In a course of routine testing of our analogs, we detected unexpectedly high stimulation of IGF-1R by [d-His^B24^]-insulin. We designed a series of new analogs to explain this observation. A schematic illustration of the analogs is shown in Fig. 3.

**Table 1:** **Receptor binding affinities of human insulin, IGF-1, IGF-2, and insulin analogs** The *K_d_* values were obtained from at least three measurements. *n* is the number of replicates. Asterisks indicate that binding of the ligand to a particular receptor differs significantly from that of insulin (*, *p* < 0.05; **, *p* < 0.01; ***, *p* < 0.001).

Analog	IR-A		IR-B		IGF-1R	
	*K_d_* ± S.D. (*n*)	Relative*^a^* binding affinity	*K_d_* ± S.D. (*n*)	Relative*^a^* binding affinity	*K_d_* ± S.D. (*n*)	Relative*^a^* binding affinity
	*nm*	%	*nm*	%	*nm*	%
Insulin*^b^*	0.36 ± 0.14^#^ (5)	100	0.55 ± 0.27^#^ (4)	100	292 ± 54.30^€^ (3)	0.08
	0.45 ± 0.11^$^ (6)		0.37 ± 0.11^$^ (4)			
	0.27 ± 0.02^∧^ (5)		0.39 ± 0.14^∧^ (4)			
	0.25 ± 0.05^≈^ (5)		0.55 ± 0.11^€^ (6)			
	0.35 ± 0.06^€^ (4)					
IGF-1*^b^*	31.50 ± 5.63^#^ (4)***	1.1	223.9 ± 32.9^#^ (4)***	0.2	0.25 ± 0.03^#^ (4)	100
					0.11 ± 0.05^$^ (5)	
					0.12 ± 0.02^∧^ (5)	
					0.24 ± 0.10^€^ (5)	
IGF-2	2.92 ± 0.24^≈^ (3)***	8.4	35.45 ± 11.22^#^ (4)***	1.6	2.32 ± 1.24^#^ (3)***	10.7
[d-His^B24^]-DTI-NH_2_	0.18 ± 0.02^#^ (3)	204	0.30 ± 0.15^#^ (4)	183	288 ± 18.61^#^ (3)	0.09
[d-His^B24^]-insulin	0.126 ± 0.015^€^ (3)**	280	0.22 ± 0.05^€^ (3)**	251	24.41 ± 10.59^#^ (3)**	1.02
Des-Phe^B24^-insulin	0.12 ± 0.01^#^ (4)*	305	0.42 ± 0.11^#^ (4)	132	77.82 ± 20.71^#^ (3)**	0.32
[Tyr^B25^, Phe^B26^, Asn^B27^, Lys^B28^, Pro^B29^]-insulin	0.31 ± 0.07^∧^ (3)	86	0.53 ± 0.21^∧^ (3)	75	143.60 ± 54.67^∧^ (5)	0.08
[Tyr^B25^, Phe^B26^, Asn^B27^, Lys^B28^, Pro^B29^, Gly^B31^, Tyr^B32^]-insulin	0.21 ± 0.06^∧^ (3)	131	0.19 ± 0.03^∧^ (3)	207	16.01 ± 6.24^∧^ (5)***	0.77
[Gly^B31^, Tyr^B32^]-insulin	0.17 ± 0.07^∧^ (3)*	162	0.13 ± 0.05^∧^ (3)*	310	45.66 ± 17.72^∧^ (4)**	0.27
[d-His^B24^, Gly^B31^, Tyr^B32^]-insulin	0.18 ± 0.02^$^ (3)**	251	0.12 ± 0.03^$^ (3)*	338	0.89 ± 0.20^$^ (6)***	12.4

*^a^* Relative binding affinity is defined as (*K_d_* of human insulin or IGF-1/*K_d_* of analog) × 100 (%).

*^b^* The *K_d_* of human insulin for IR-A was determined in five independent measurements (#, $, ∧, ≈, and €) and for IR-B in four measurements (#, $, ∧, and €), and *K_d_* of human IGF-1 for IGF-1R was determined in four measurements (#, $, ∧, and €). The individual values of *K_d_* of a particular ligand are relative to a corresponding insulin or IGF-1 *K_d_* values (e.g. # to #, etc.).

First, we aimed to check whether the d-His residue has any influence on IGF-1 receptor binding (in [d-His^B24^]-DTI-NH_2_). Next, we examined the relevance of a downshift of Phe^B25^ to the position of Phe^B24^ and, in parallel, the switch of Lys^B29^ and Pro^B28^, which corresponds to the sequence in IGF-1 (in des-Phe^B24^-insulin). We assumed that [d-His^B24^]-insulin can adopt structural features mimicking respective parts of the IGF-1 B and C domains. Thus, we employed the B domain C-terminal sequence of IGF-1 (in [Tyr^B25^, Phe^B26^, Asn^B27^, Lys^B28^, Pro^B29^]-insulin) and also extended the C terminus of insulin analogs with Gly^B31^-Tyr^B32^ residues, because Tyr^31^ in IGF-1 was shown to be important for IGF-1 receptor selectivity and activation (30) (in [Tyr^B25^, Phe^B26^, Asn^B27^, Lys^B28^, Pro^B29^, Gly^B31^, Tyr^B32^]-insulin and in [Gly^B31^, Tyr^B32^]-insulin). Finally, [d-His^B24^, Gly^B31^, Tyr^B32^]-insulin was designed to combine several tested features.

### NMR spectroscopy and structure of [d-His^B24^, Gly^B31^, Tyr^B32^]-insulin

The NMR spectra of [d-His^B24^, Gly^B31^, Tyr^B32^]-insulin were acquired under the same conditions as used for [d-His^B24^]-insulin described previously (29). The comparison of proton NMR data for both insulin analogs showed nearly the same values of chemical shifts (Δδ < 0.05 ppm) for protons of all residues except Lys^B29^ and Thr^B30^. This is not surprising because they are the last two B chain residues in [d-His^B24^]-insulin, whereas in the [d-His^B24^, Gly^B31^, Tyr^B32^]-insulin, the chemical shifts of Lys^B29^ and Thr^B30^ are somewhat influenced by subsequent Gly^31^ and Tyr^32^. A comparison of the chemical shifts of backbone NH and Hα protons is presented in Table S2 and graphically demonstrated in Fig. S1. Based on these data, we can conclude that solution NMR structure of [d-His^B24^, Gly^B31^, Tyr^B32^]-insulin, except residues B29–B32, does not differ from the structure of [d-His^B24^]-insulin described previously. The hydrophobic pocket of Phe^B24^ was filled with Phe^B25^, and d-His^B24^ was left protruding from the structure. Tyr^B26^ replaced the position of Phe^B25^, and residues B27–B32 departed from the insulin core, thus mimicking the previously observed downshift in [d-His^B24^]-insulin. The same conclusions for [d-His^B24^, Gly^B31^, Tyr^B32^] are supported by the presence of NOE cross-peaks Tyr^B16^Hα/Phe^B25^Hδ, Hϵ, Hζ as well as Phe^B25^Hϵ/Tyr^B16^Hδ, Hϵ cross-peaks. The absence of any medium- or long-range NOEs indicates a high degree of flexibility of the B26–B32 part.

### Binding and stimulation of IR-A and IR-B

**Figure 4.**
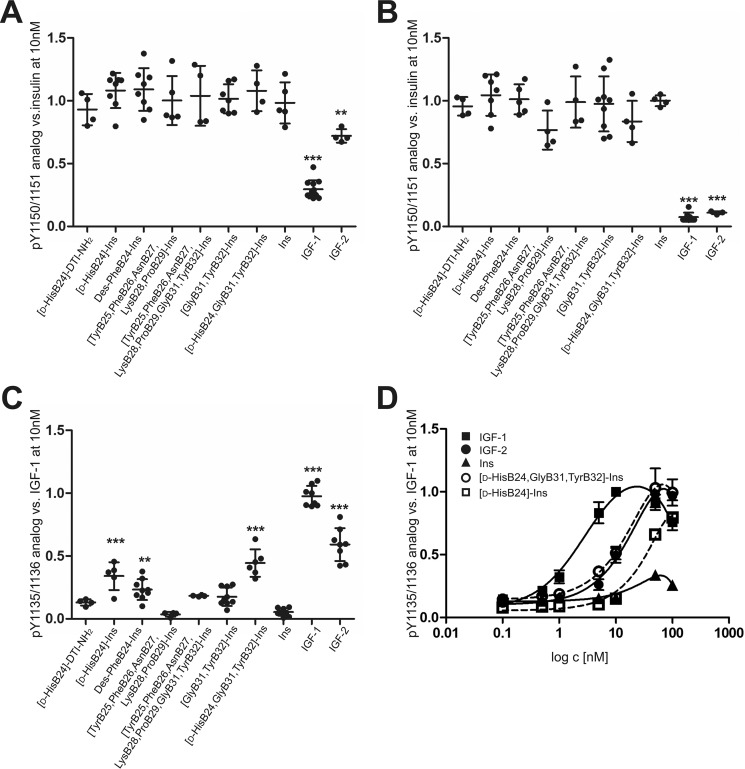
**Relative abilities of human insulin, IGF-1, IGF-2, and insulin analogs to stimulate receptor phosphorylation.** IR-A–transfected cells (*A*), IR-B–transfected cells (*B*), and IGF-1R–transfected cells (*C*) were stimulated with 10 nm ligands for 10 min. *D*, IGF-1R–transfected cells were stimulated with a 0.1–100 nm concentration range of WT ligands (*continuous lines*) and selected analogs (*dashed lines*). The data (mean ± S.D. (*error bars*), *n* ≥ *4*) were expressed as the contribution of phosphorylation relative to the signal of human insulin (IR-A, IR-B) or IGF-1 (IGF-1R) at 10 nm in the same experiment. Data in *A–C* are from immunoblotting, and data in *D* were obtained using the In-Cell Western assay. Representative blots are shown in supporting Figs. S6 and S7. *Ins*, human insulin. *Asterisks* indicate that phosphorylation of the receptor induced by a ligand differs significantly from that of insulin (*, *p* < 0.05; **, *p* < 0.01; ***, *p* < 0.001).

Five of seven analogs bound both IR-A and IR-B with increased affinity compared with human insulin (Table 1 and Figs. S2–S4). [Tyr^B25^, Phe^B26^, Asn^B27^, Lys^B28^, Pro^B29^]-insulin and [Tyr^B25^, Phe^B26^, Asn^B27^, Lys^B28^, Pro^B29^, Gly^B31^, Tyr^B32^]-insulin, which bears the C-terminal sequence of the IGF-1 B domain, had affinity similar to insulin. The analogs with an extended C terminus with Gly^B31^–Tyr^B32^ showed an increased ratio in IR-A/IR-B binding in favor of IR-B, whereas shortened analog des-Phe^B24^-insulin showed the inverse ratio. We did not detect any deviations in the abilities of the analogs to stimulate the receptors compared with insulin (Fig. 4 (*A* and *B*) and Fig. S6).

### Binding and stimulation of IGF-1R

The binding affinities (Table 1) of two analogs toward IGF-1R as well as their ability to stimulate the receptorwere unchanged compared with insulin. [d-His^B24^]-DTI-NH_2_ bound IGF-1R equally to insulin. Also, [Tyr^B25^, Phe^B26^, Asn^B27^, Lys^B28^, Pro^B29^]-insulin, which bears the C-terminal sequence of the IGF-1 B domain, showed no change.

The addition of Gly^B31^-Tyr^B32^ to insulin increased binding to IGF-1R about 3-fold, and the change of the sequence B23-B32 to an IGF-1–like sequence even increased binding 9-fold, although both analogs stimulated autophosphorylation of IGF-1R at 10 nm concentration similarly (Fig. 4*C*). The stimulation was increased about 3-fold compared with insulin, but the change appeared not to be significant in the analysis of variance.

Des-Phe^B24^-insulin, [d-His^B24^]-insulin, and [d-His^B24^, Gly^B31^,Tyr^B32^]-insulin showed enhanced binding to IGF-1R, accompanied with increased stimulation of the receptor. Binding of [d-His^B24^, Gly^B31^, Tyr^B32^]-insulin to IGF-1R was increased about 150-fold compared with insulin and was comparable with the IGF-2 binding. The analogs [d-His^B24^]-insulin and [d-His^B24^, Gly^B31^, Tyr^B32^]-insulin were able to stimulate the IGF-1R at a 10 nm concentration to an extent approximating IGF-2 (Fig. 4*C* and Fig. S6). The dose–response curves of IGF-1R stimulation (Fig. 4*D* and Fig. S7) showed that [d-His^B24^, Gly^B31^, Tyr^B32^]-insulin is equipotent to IGF-2 and that [d-His^B24^]-insulin is a less effective activator of IGF-1R than IGF-2, but significantly stronger than human insulin. In our further experiments, we focused on the most interesting analog, [d-His^B24^, Gly^B31^, Tyr^B32^]-insulin.

### Binding kinetics

**Figure 5.**
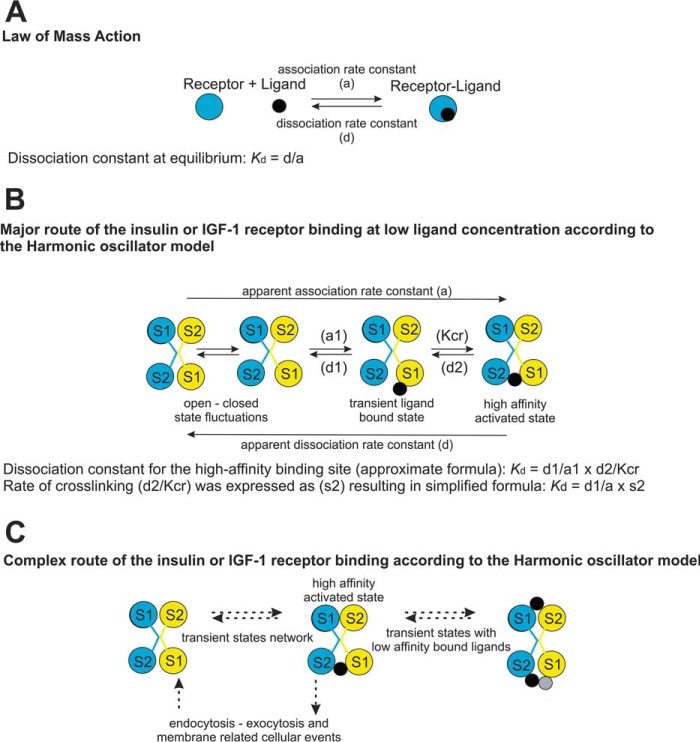
**Simplified scheme of receptor–ligand interaction models used for interpretation of kinetic measurements.** *A*, the law of mass action model; *B*, harmonic oscillator model, adapted from (6, 7) of insulin or IGF-1 binding at low ligand concentration, where *a1* is the association and *d1* is the dissociation coefficient of Site 1 (*S1*); *d2* is the dissociation coefficient of Site 2 (*S2*), and *Kcr* is a constant characterizing the Site 1 and Site 2 assembly to form the high-affinity complex. *C*, model of insulin or IGF-1 receptor binding. Multiple factors influencing the kinetics are suggested. Receptors are represented by a *blue circle* or an assembly of *blue* and *yellow circles* showing binding sites on IR or IGF-1R homodimers. *Black circles*, ligands; *gray circle*, third ligand molecule bound to the IR, which is not allowed on IGF-1R.

To interpret the data, we applied the presumptions and relations from the harmonic oscillator model (7, 31), as explained under “Experimental Procedures.” The model is schematically presented in Fig. 5. We considered only the major route (Fig. 5*B*) of a ligand cross-linking two binding sites on the receptor and ignored the other events, such as the rate of endocytosis and multiple low-affinity binding states (Fig. 5*C*).

**Figure 6.**
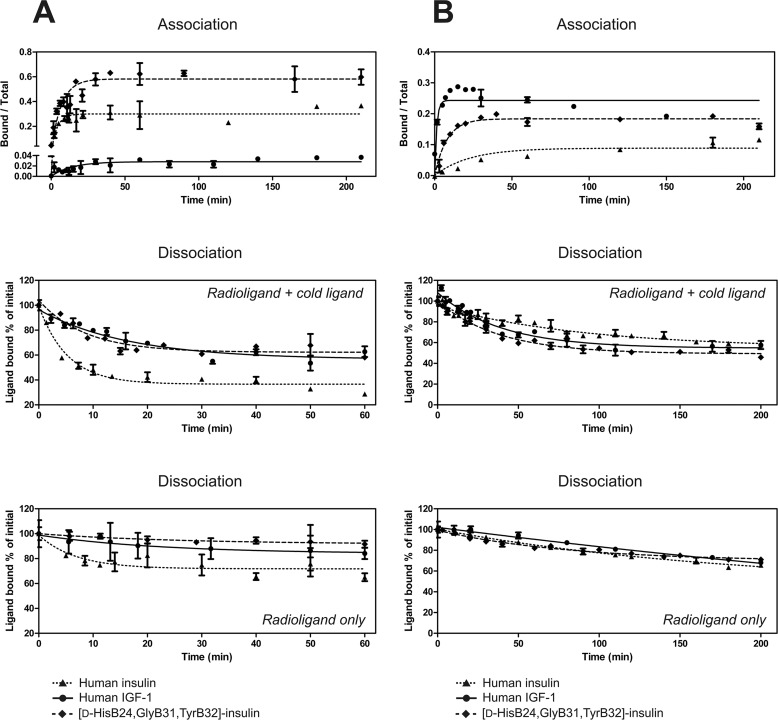
**Association and dissociation assays.** *A* and *B*, association and dissociation of human ^125^I-insulin, ^125^I-IGF-1, and ^125^I-[d-His^B24^, Gly^B31^, Tyr^B32^]-insulin from IR-A in human IM-9 cells (*A*) and from IGF-1R in the CHO-K1 cell line stably transfected with human IGF-1R (*B*). Data are the mean ± S.D. (*error bars*) of duplicate values of a representative experiment. The results are expressed as the ^125^I-labeled ligand bound at the specific time point over total. Dissociation was measured in the presence of unlabeled ligand (for details, see “Experimental Procedures”) and with the radioligand only. Results are expressed as the percentage of ^125^I-labeled ligand bound at *t* = 0. Representative curves are shown.

We measured association and dissociation kinetics (Fig. 6) and dose–response curves for accelerated dissociation of ^125^I-labeled insulin, IGF-1, and [d-His^B24^, Gly^B31^, Tyr^B32^]-insulin toward the IR-A and IGF-1R.

Dose–response curves for accelerated dissociation had the same shape as reported previously (7) (*i.e.* bell-shaped for IR-A and sigmoid for IGF-1R in the case of all three ligands: human insulin, IGF-1, and [d-His^B24^, Gly^B31^, Tyr^B32^]-insulin) (Fig. S5).

To interpret the association and dissociation kinetics (Fig. 6), the measured *K_d_* ratios (Table 1) were related to the ratios of dissociation coefficient at maximal acceleration, which should depend solely on Site 1 interactions (*d1*) (Fig. 5*B*) (31). In cases where the difference in interaction with receptor between ligands is dependent mainly on Site 1 interaction, the ratios of measured *K_d_* and the *d1* and *a1* constants will follow a simple formula where *X* represents one ligand and *Y* is the other.

(Eq. 1) $$K_{d}X/K_{d}Y=d1X/d1Y\times a1Y/a1X$$

We compared the measured apparent association constants (*a*) with theoretically expected values for Site 1 (*a1*) (Fig. 5*B*) and investigated whether the factors related to the Site 1–Site 2 (*s2*) cross-linking reaction must be encompassed by the following equation.

(Eq. 2) $$K_{d}X/K_{d}Y=d1X/d1Y\times aY/aX\times s2X/s2Y$$

The discussed ratios of the coefficients are shown in Table 2. The source data are in Table S1. Although the measurements showed high variability evident from the S.D. values (Table S1) and deviations from standard shape of the curves (Fig. 6*B*, IGF-1 association) we can draw the following conclusions.

**Table 2:** **Approximate ratios of kinetics factors for interaction of human insulin, IGF-1, and [d-His^B24^, Gly^B31^, Tyr^B32^]-insulin (An) with IR-A and IGF-1R** Ratios were calculated from mean values of the measured parameters. Relative S.D. values were in the range 10–50%; thus, the ratios must be considered as approximate. *X* and *Y* represent the values for the ligands listed in individual columns (insulin, IGF-1, and [d-His^B24^, Gly^B31^, Tyr^B32^]). For details, see “Experimental procedures” and Table S1. An, [d-His^B24^, Gly^B31^, Tyr^B32^]-insulin.

Receptor type	Parameter	Insulin/IGF-1	Insulin/An	IGF-1/An
IR-A	*K_d_**^a^*	1:88	2.5:1	221:1
	*d1**^b^*	6.4: 1	3.4: 1	1: 1.9
	Theor. *a1X*/*a1Y* =	563: 1	1.4: 1	1: 420
	*d1X*/*d1Y* × *K_d_Y/K_d_X**^c^*			
	*a*_meas._*^d^*	1.1: 1	1: 1.1	1: 1.3
	*a*_derived_	1.6: 1	1.2: 1	1: 1.3
	Factor site 2 *s2X*/*s2Y* =	1: 512	1: 1.5	323: 1
	*K_d_X/K_d_Y* × *aX/aY* × *d1Y/d1X**^e^*	1: 352	1: 1.1	323: 1
IGF-1R	*K_d_**^a^*	1217: 1	150: 1	1: 8
	*d1**^b^*	1: 3	1: 3	1: 1
	Theor. *a1X*/*a1Y* =	1: 3651	1: 479	8: 1
	*d1X*/*d1Y* × *K_d_Y/K_d_X**^c^*			
	*a*_meas._*^d^*	1: 100	1: 15	6.7: 1
	*a*_derived_	1: 89	1: 14	6.4: 1
	Factor site 2 *s2X*/*s2Y* =	37: 1	30: 1	1: 1.2
	*K_d_X/K_d_Y* × *aX/aY* × *d1Y/d1X**^e^*	41: 1	32: 1	1: 1.3

*^a^* Dissociation constants (*K_d_*) are from receptor-binding assays.

*^b^* Ratios of dissociation factors for Site 1 (*d1*) were derived from the dissociation rate at maximal acceleration.

*^c^* Theoretical ratios of association constants (Theor. *a1*) were calculated as if the reaction followed the simple model of the law of mass action.

*^d^* Association factor (*a*) was derived from experimental curves, using the measured rate of dissociation of ^125^I-labeled ligand without the presence of cold ligand (*a*_meas._), or constants calculated in the model for negative cooperativity (6) were applied (*a*_derived_).

*^e^* Factor Site 2 (*s2*) is an estimated value to complement the equation, using both association factors (*a*_meas._ upper value and *a*_derived_ lower value).

Association of all three ligands (insulin, IGF-1, and [d-His^B24^, Gly^B31^, Tyr^B32^]-insulin with IR-A was very fast, whereas dissociation of IGF-1 and [d-His^B24^, Gly^B31^, Tyr^B32^]-insulin was markedly slower than that of insulin (Fig. 6*A*). In the case of [d-His^B24^, Gly^B31^, Tyr^B32^]-insulin, the decrease of the dissociation rate (*d1*) (3.4-fold) was reflected by an increase in *K_d_* (2.5-fold) (Table 2). Our data confirm the presumption that the difference in *K_d_* values between [d-His^B24^, Gly^B31^, Tyr^B32^]-insulin and insulin is related to the Site 1 interaction.

In the case of IGF-1 binding to IR-A, it was evident that factors other than simple interaction with Site 1 must play a role. If a simple model of law of mass action (Fig. 5*A*) is applied, the expected ratio of association coefficients (*a*) is 563:1 (association of IGF-1 with IR-A would be 563-fold slower than with insulin). However, the determined ratio was ∼1:1 (Table 2). The factors driving interaction with Site 2 (*s2*) are expected to play a major role in binding of insulin to IR-A, compared with binding of IGF-1.

The situation was opposite in the case of binding of the ligands to IGF-1R. Interaction of insulin with IGF-1R was characterized by a very slow association rate, whereas the dissociation rate was similar to that of IGF-1 (Fig. 6*B*). The factors driving interaction with Site 2 (*s2*) are also expected to be changed, but not to as great an extent as in the case of IGF-1 interacting with IR-A (∼1:400 in favor of insulin binding to IR-A, compared with 1:40 in favor of IGF-1 binding to IGF-1R) (Table 2). Increase in the association rate (about 15-fold) contributed substantially to the increase in *K_d_* for IGF-1R of the [d-His^B24^, Gly^B31^, Tyr^B32^]-insulin compared with insulin (Fig. 6 and Table 2).

## Discussion

Our laboratory is interested in the design of insulin and IGFs analogs, which should map the structure–activity relationship among ligands and their receptors and potentially serve medicinal purposes (22, 23, 29, 32–35). In the course of routine testing of our analogs, we have detected the unexpectedly high binding and stimulation of IGF-1R by [d-His^B24^]-insulin (29). The binding and activation of IGF-1R was further dramatically accelerated by the addition of Gly^31^-Tyr^32^ to the C terminus of the B chain (251 or 338% binding affinity to IR-A respective to IR-B relative to insulin and 12.4% binding affinity to IGF-1R relative to IGF-1). To the best of our knowledge, the analog [d-His^B24^, Gly^B31^, Tyr^B32^]-insulin is one of the strongest insulin-like binders and activators of IGF-1R thus far reported and is a significantly more potent IGF-1R binder than the well-known “supermitogenic” analog X10 ([Asp^B10^]-insulin) or a strong binder [Arg^B31^, Arg^B32^]-insulin (26, 36). Maybe only a chimera where insulin A and B chains are connected by the C-loop of IGF-1 is a stronger binder of IGF-1R (9). The chimera bound IR with 113% affinity relative to insulin and IGF-1R with 28% relative to IGF-1. However, [d-His^B24^, Gly^B31^, Tyr^B32^]-insulin differs from human insulin only in three positions but the chimera differs from human insulin in 12 residues (the whole extra C domain).

Among our analogs, [d-His^B24^]-DTI-NH_2_ was primarily prepared to check whether the d-His residue exposed out of the insulin core has any influence on IGF-1R stimulation. Generally, des-(B27-B30)-B26-carboxamide insulins (-DTI-NH_2_) are characterized by high affinity to the IR (22, 23). This affinity has been explained by exposing the hydrophobic residues A1–A3 to direct interaction with the receptor. We tested the ability of other -DTI-NH_2_ insulin analogs to stimulate IGF-1R ([*N*-MeAla^B26^]-DTI-NH_2_ and [d-Pro^B26^]-DTI-NH_2_ (23); data not shown). The shortened analogs had characteristics comparable with the [d-His^B24^]-DTI-NH_2_. The ability to bind and activate IGF-1R was similar to that of insulin. We concluded that the d-HisB24 residue and its structural effect on adjacent B25 and B26 positions makes no contribution to the increased IGF-1R binding and activation. Exposing the residues A1–A3 might have a certain impact on increased affinity to the IR, but not to the IGF-1R.

Other analogs [Tyr^B25^, Phe^B26^, Asn^B27^, Lys^B28^, Pro^B29^]-insulin, [Tyr^B25^, Phe^B26^, Asn^B27^, Lys^B28^, Pro^B29^, Gly^B31^, Tyr^B32^]-insulin, and [Gly^B31^, Tyr^B32^]-insulin were designed to probe whether the adoption of the IGF-1–like sequence in this portion of the molecule has any effect on binding and activation of the receptors. The [Tyr^B25^, Phe^B26^, Asn^B27^, Lys^B28^, Pro^B29^]-insulin did not acquire any new quality. The extension of the sequences by Gly^B31^-Tyr^B32^ increased the affinity and activity of these analogs toward the IGF-1R. Surprisingly, the addition of Gly^B31^-Tyr^B32^ also improved binding to IR-A and even more profoundly to IR-B. These results do not fit with the presumption that Tyr^31^ contributes to IGF-1R selectivity (30).

We also studied a previously reported des-Phe^B24^-insulin (37). We synthetized this analog to prove the relevance of downshift of Phe^B25^ to the position of Phe^B24^. Our expectations were only partially complied with. This analog showed a high affinity to the IR-A and increased binding and activation of IGF-1R. The high affinity of des-Phe^B24^-insulin to IR was explained analogously to the shortened -DTI-NH_2_- insulins and [d-His^B24^]-insulin (*i.e.* by exposing A1-A3 residues, due to the relaxed structure at the C terminus of the B chain) (37). However, based on our data, the simple uncovering of the A1–A3 region is not the most likely driving force for an increased affinity of the analogs to the IGF-1R. We rather consider the idea that the specific change in the structure of the C-terminal part of the B chain in [d-His^B24^, Gly^B31^, Tyr^B32^]-insulin, resulting in its relaxation and redirection (in analogy with [d-His^B24^]-insulin as confirmed here by NMR analysis), allows the residues to use a hidden potential of the receptor and bind it by means of some uncommon contacts.

To obtain a closer insight into the binding characteristics of this analog, we performed measurements of association and dissociation kinetics of insulin, IGF-1, and the [d-His^B24^, Gly^B31^, Tyr^B32^]-insulin toward the IR and IGF-1R (Fig. 6). We are aware that the presumptions used to interpret the data and our estimate calculations are roughly simplistic, but the conclusions seem to be eloquent. The presumption that IR and IGF-1R differ in their activation mechanism was supported. The difference between the receptors has already been implied from the shapes of the curves for negative cooperativity (7), from different binding characteristics of solubilized receptors compared with the membrane-bound receptors (5), and recently also from the structural studies (6, 12).

Based on our data, factors related to the formation of the high-affinity complex and dissociation kinetics seem to play the major role in the different affinities of the ligands (insulin, IGF-1, and the analog) for IR-A. The dissociation kinetics would probably be an even more distinguishing factor for IR-B affinity. We observed previously that specific extension of the C terminus of the insulin B chain can increase the affinity to IR-B (33). This is probably caused by additional contacts of the prolonged B chain of analogs with the receptor that slow down the dissociation from the receptor, similarly to our analogs with Gly^B31^-Tyr^B32^.

Conversely, association kinetics seems to be the main factor in IGF-1R affinity. Site 1 on the receptors involves the N terminus of the L1 domain of one receptor α subunit and C-terminal residues of the insert domain of a second α subunit (αCT peptide). Site 1 on IR interacts with insulin amino acid residues: Gly^A1^, Ile^A2^, Val^A3^, Glu^A4^, Tyr^A19^, Gly^B8^, Ser^B9^, Leu^B11^, Val^B12^, Tyr^B16^, Phe^B24^, Phe^B25^, and Tyr^B26^ (8). Identical or homologous residues are present at the same positions in IGF-1 (Fig. 1) and were shown to interact with the IR as well as with IGF-1R in a similar manner (6, 12, 13). The only differences are in Tyr^B16^ and Ser^B9^ in insulin, which are replaced with Gln^15^ and Ala^8^ in IGF-1, respectively. In addition, Met^59^ in IGF-1, which does not have equivalent receptor-binding residue in insulin, was shown to interact with Arg^704^ IGF-1R αCT peptide, and its mutation abolishes receptor binding (34). Ala^8^ in IGF-1 was shown to interact with both the L1 domain (Glu^91^) and αCT peptide (Glu^693^) of IGF-1R, whereas insulin Ser^B9^ interacts only with the IR αCT peptide (His^710^) (6, 8). Insulin Tyr^B16^ interacts with Phe^39^ of IR L1 domain, which corresponds to Ser^35^ in IGF-1R, which is not involved in ligand binding (8, 28). Accordingly, the interaction of insulin Tyr^B16^ with receptor Phe^39^ is crucial for specificity of insulin binding to IR (specificity-conferring region 1–68 IR) (9). We hypothesize that the lack of interactions provided by Tyr^B16^ in insulin prevents anchoring of insulin on IGF-1R and accordingly slows down the association. We speculate that relaxation of the C terminus and the addition of Gly^B31^-Tyr^B32^ in the [d-His^B24^, Gly^B31^, Tyr^B32^]-insulin provide additional contacts with the receptor, not necessarily the same as in IGF-1, and partially compensate for the missing anchor. We do not think that mimicking of structural features of the IGF-1 C-loop is the main reason for increased binding of [d-His^B24^, Gly^B31^, Tyr^B32^]-insulin to IGF-1R, because then also the other analogs with Gly^B31^-Tyr^B32^ and not [d-His^B24^]-insulin would have the same characteristics.

Phylogeny of insulin-like peptides and their interacting partners (receptors and binding proteins) dates to an outset of the Animalia kingdom. Insulin-like peptides can be found in primitive deuterostomes (38) as well as in insects (39). Whereas there is a vast diversity of insulin-like peptides sharing a similar fold of a compact three-dimensional structure, only one type of insulin-like receptor is found in the lower species. The separate IR and IGF-1R do not appear sooner than in vertebrates, and insulin receptor isoforms IR-A and IR-B exist only in mammals (2). The structural origins of the selectivity of the ligands (insulin, IGF-1, and IGF-2) to their cognate receptors still remain a mystery and a great example of natural selection. On the other hand, it is tempting to presume that the multiple insulin-like peptides found in invertebrate species should have the ability to exert specific functions, even though they act through the same receptor. Eliciting of different signaling and biological responses on the same receptor through the action of different ligands (insulin, IGFs or mimetic peptide S597) was reported (40, 41). The molecular mechanisms responsible for how different ligands activating the same receptor can initiate different biological responses in the same cell are not completely understood (42). It is thus possible that there is more than one way of activating the receptor. Our data with [d-His^B24^, Gly^B31^, Tyr^B32^]-insulin supports this assumption.

Based on the course of binding of insulin, IGF-1, and [d-His^B24^, Gly^B31^, Tyr^B32^]-insulin to the receptors, we offer insight into the factors contributing to the selectivity to the receptors. These discoveries can provide clues for the design of selective analogs and possibly antagonists of the receptors and demonstrate the power and effectivity of rational hormone engineering.

## Experimental procedures

### Synthesis of analogs

Previously described insulin analog [d-His^B24^]-insulin (29) and five newly prepared analogs ([d-His^B24^]-DTI-NH_2_, [Tyr^B25^, Phe^B26^, Asn^B27^, Lys^B28^, Pro^B29^]-insulin, [Tyr^B25^, Phe^B26^, Asn^B27^, Lys^B28^, Pro^B29^, Gly^B31^, Tyr^B32^]-insulin, [Gly^B31^, Tyr^B32^]-insulin, and [d-His^B24^, Gly^B31^, Tyr^B32^]-insulin), together with des-Phe^B24^-insulin (37), were prepared by enzymatic semisynthesis of des-(B23-B30)-octapeptide insulin and corresponding tetra-, hepta-, octa-, and decapeptides. All semisynthetic procedures were described in detail previously (22), except that we used Fmoc-Lys(Pac)-OH, which was prepared by a modification (see supporting information) of a method described previously (43). The identities of peptides and insulin analogs were confirmed, using mass spectrometer LTQ-orbitrap XL (Thermo Fisher) or the TripleTOF^TM^ 5600 system (AB SCIEX), and their purities (≥95%) were controlled by analytical HPLC. A schematic presentation of the analogs is shown in Fig. 3.

### NMR spectroscopy

NMR spectra of 0.2 mm nonlabeled [d-His^B24^, Gly^B31^, Tyr^B32^]-insulin were acquired as a 0.4-ml solution in H_2_O + D_2_O (95:5) with 25 mm deuterated Tris buffer (pH 8.0) at 25 °C on a 600-MHz Bruker AVANCE spectrometer equipped with a triple-resonance cryoprobe. A series of 2D homonuclear spectra was recorded for structural assignment of proton signals: 2D TOCSY spectra with a mixing time 30, 60, and 90 ms and 2D NOESY spectra with mixing time 150, 200, and 300 ms. Proton NMR data are shown in Table S3.

### Cell culture

Human IM-9 lymphocytes (ATCC) and mouse embryonic fibroblasts (IR-A, IR-B, and R^+39^) derived from IGF-1R knockout mice and stably transfected with the receptors IR-A, IR-B, and IGF-1R, kindly provided by A. Belfiore (Catanzarro, Italy) and R. Baserga (Philadelphia, PA), were grown as described previously (33, 42).

Chinese hamster ovary cell line CHO-K1 (ATCC) was stably transfected with pcDNA3-IGF-1R vector, kindly provided by R. O'Connor (Cork, Ireland), using Lipofectamine 2000 reagent and Geneticin^TM^ (Thermo Fisher Scientific) as a selection antibiotic. A polyclonal population of cells stably expressing human IGF-1R was obtained (CCHO-R+). The cells were grown in Ham's F-12 medium, supplemented with 10% fetal bovine serum, 2 mm l-glutamine, 0.5 mg/ml Geneticin, 100 units/ml penicillin, and 100 μg/ml streptomycin in humidified air with 5% CO_2_ at 37 °C. For receptor binding studies, the cells were trypsinized and transferred to an Erlenmeyer flask (5 × 10^6^ cells/ml). They were maintained floating by constant agitation at 140 rpm at 37 °C overnight.

### Receptor-binding studies

Human IM-9 lymphocytes (containing IR-A) and IGF-1R null mouse embryonic fibroblasts, stably transfected with either human IR-B or human IGF-1R, were employed for a whole-cell receptor-binding assay as described previously (32, 33, 42). For details, see supporting information. The binding curve of each analog was determined in duplicate, and the final dissociation constant (*K_d_*) was calculated from at least three (*n* ≥ 3) binding curves. Significance of the changes in binding affinities in relation to insulin or IGF-1 was calculated using the two-tailed *t* test.

Dose–response curves for negative cooperativity (accelerated dissociation), association kinetics, and dissociation kinetics were measured for insulin, IGF-1, and [d-His^B24^, Gly^B31^, Tyr^B32^]-insulin toward the IR-A and IGF-1R. Basically, the procedures followed the published protocols (44). For details, see supporting information. Each curve was determined in duplicate, and experiments were repeated at least twice. IM-9 cells were used for IR-A measurements, and CHO-R+ cells were used for IGF-1R.

Labeled mono-^125^I-insulin and mono-^125^I-IGF-1 were purchased from PerkinElmer Life Sciences. Iodination of [d-His^B24^, Gly^B31^, Tyr^B32^]-insulin with ^125^I (Na[^125^I], product code I-RB-41, 0.1 mCi; Izotóp Intézet Kft. (Budapest, Hungary)) was performed, using the IODO-GEN^TM^ system (Pierce). The mono-iodinated ligand was separated on a Nucleosil 120 C18 column (5μ, 250 × 4.0 mm; Macherey Nagel). A detailed description of the iodination procedure is included in the supporting Methods.

For accelerated dissociation, the cells were preincubated with radiolabeled ligand for 2.5 h at 15 °C in all cases. For dissociation kinetics, the preincubation duration was estimated based on association kinetics (time after reaching the steady state). The cells were preincubated with radiolabeled ligand for 2.5 h in the case of insulin and analog binding to IR-A. In the case of IGF-1 binding to IR-A, the preincubation lasted 4 h. The period was 2.5 h for analog and IGF-1 binding to IGF-1R, and 7 h in the case of insulin binding to IGF-1R.

Dissociation kinetics of a radiolabeled ligand was determined both without and in the presence of cold ligand (170 nm for all of the experiments, except for insulin on IGF-1R, where a 17 μm solution of insulin was used).

### Binding kinetics

To interpret the data obtained in the course of measuring the association and dissociation kinetics, we adopted some presumptions and relations from the harmonic oscillator model of insulin binding (7, 31) (Fig. 5).

#### 

##### First presumption

*K_d_* value for the apparent high-affinity binding site can be calculated according to an approximate formula,

(Eq. 3) $$K_{d}=d1/a1\times d2/Kcr$$

where *a1* is an association and *d1* is a dissociation coefficient of Site 1; *d2* is the dissociation coefficient of Site 2, and *Kcr* is a constant characterizing the Site 1 and Site 2 assembly to form the high-affinity complex.

##### Second presumption

Dissociation rate at maximum acceleration is proportional to the dissociation coefficient for Site 1 (*d1*).

##### Third presumption

If the difference in interaction with receptor between ligands is dependent solely on Site 1 interaction, then the ratios of measured *K_d_* and *d1* will be proportional to a ratio of constants (*a1*) of one ligand (*X*) to the other ligand (*Y*) as in Equation 1.

If the kinetics of the Site 1–Site 2 assembly is influencing the interaction, the found ratios of association coefficients will be disproportional and dependent on the Site 2 coefficients as follows.

(Eq. 4) $$k_{d}X/K_{d}Y=d1X/d1Y\times a1Y/a1X\times d2X/d2Y\times Kcr2Y/Kcr2X$$

Because we cannot estimate the *d2* and *Kcr*, we have simplified the formula to Equation 2, where *s2* is a factor driven by the Site 2 interactions, and *a* is an apparent association coefficient determined from the experimental data.

The dissociation constants were calculated after fitting the experimental data to monoexponential decay in GraphPad Prism version 5. To fit experimental data to association kinetics in GraphPad Prism 5, it is mandatory to set the dissociation constant. However, assignment of this is ambiguous due to a multistep process of ligand binding and concentration dependence. Thus, the determined association and dissociation constants should be considered artificial. For calculations, we used the measured constants determined from dissociation in the absence of cold ligand. We also used constants calculated for first ligand dissociation (condition of low ligand concentration), derived from a recently refined model for negative cooperativity (6). The constants were adjusted to the respective analog according to the found *d1* ratios. The data are shown in Table S1.

### Receptor phosphorylation assay

Cell stimulation and detection of receptor phosphorylation were performed as described previously (33), using mouse fibroblasts (IR-A, IR-B, and R^+39^). For details, see supporting information. The cells were stimulated with 10 nm concentrations of the ligands for 10 min. Proteins were routinely analyzed using immunoblotting. The membranes were probed with anti-phospho-IGF-1Rβ (Tyr^1135/1136^)/IRβ (Tyr^1150/1151^) (Cell Signaling Technology). Each experiment was repeated four times. The data were expressed as the contribution of phosphorylation relative to the human insulin (IR-A, IR-B) or IGF-1 (IGF-1R) signal in the same experiment. Mean ± S.D. (*n* ≥ 4) values were calculated. The significance of the changes in stimulation of phosphorylation in relation to insulin was calculated, using one-way analysis of variance with Dunnett's test comparing all analogs *versus* control (*i.e.* insulin).

Ligand–dose response IGF-1R autophosphorylation levels for selected analogs were determined, using the In-Cell Western assay adapted for chemiluminescence as described (34). Data were subtracted from background values and expressed as the contribution of phosphorylation relative to the 10 nm IGF-1 signal. Experiments were repeated at least four times. Log(agonist) *versus* response curve fitting of data was carried out with GraphPad Prism 5.

## Author contributions

M. C., L. Ž., A. M., O. S., M. B., and I. S. data curation; M. C., L. Ž., M. H., J. P., K. M., and I. S. investigation; M. C., L. Ž., A. M., O. S., M. B., M. H., J. P., K. M., and I. S. methodology; J. J. and I. S. conceptualization; J. J. funding acquisition; J. J. and I. S. writing-original draft; I. S. supervision.

## Supplementary Material

Supporting Information

## Footnotes

3 The abbreviations used are:

IGF: insulin-like growth factor
IR: insulin receptor
CR: cysteine-rich
IGF-1R: IGF-1 receptor
Fmoc: *N*-(9-fluorenyl)methoxycarbonyl
2D: two-dimensional
PDB: Protein Data Bank.
