# Supplementary material for: A versatile insulin analog with high potency for both insulin and insulin-like growth factor 1 receptors: Structural implications for receptor binding
Source: J Biol Chem. 2018 Sep 13;293(43):16818–29. doi: 10.1074/jbc.RA118.004852 (PMC6204900; doi:10.1074/jbc.RA118.004852)
Supplement: Supporting Information [file supp_293_43_16818__index.html]

A versatile insulin analog with high potency for both insulin and insulin-like growth factor 1 receptors: Structural implications for receptor binding — Receptor - versatile insulin analog — A versatile insulin analog with high potency for both insulin and insulin-like growth factor 1 receptors: Structural implications for receptor binding — Receptor-versatile insulin analog — Supporting Information 

# A versatile insulin analog with high potency for both insulin and insulin-like growth factor 1 receptors: Structural implications for receptor binding

## Supporting Information

- Supporting Information (to be published online) - Supporting Information for Chrudinova et al.
