# Supplementary material for: A versatile insulin analog with high potency for both insulin and insulin-like growth factor 1 receptors: Structural implications for receptor binding
Source: J Biol Chem. 2018 Sep 13;293(43):16818–29. doi: 10.1074/jbc.RA118.004852 (PMC6204900; doi:10.1074/jbc.RA118.004852)
Supplement: Supporting Information [file supp_RA118.004852_139387_1_supp_200301_psw6xs.pdf]

Supporting Information

for

A versatile insulin analog with high potency for both insulin and insulin-like growth factor 1 receptors: Structural implications for receptor binding

Martina Chrudinová, Lenka Žáková, Aleš Marek, Ondřej Socha, Miloš Buděšínský, Martin Hubálek, Jan Pícha, Kateřina Macháčková, Jiří Jiráček and Irena Selicharová

Table of Contents

Supplementary Methods

Methodology for the synthesis of Fmoc-L-Lys(Pac)-OH for peptide synthesis Pages S2-S4

Preparation of ^{125}I-[D-HisB24, GlyB31, TyrB32]-insulin Pages S4-S6

Receptor-binding Studies (in detail) Pages S6-S8

Receptor Phosphorylation Assay (in detail) Pages S8-S9

Supplementary Tables

Table S1. Source data for calculation of kinetics coefficient ratios Pages S10-S11

Table S2. Chemical shift differences of backbone HN and Hα protons Page S12

Table S3. Proton NMR data Page S13

Supplementary Figures

Figure S1. Graphical presentation of chemical shift differences Page S14

Figure S2. Inhibition of binding of human ^{125}I -Insulin to human IR-A by analogs Page S15

Figure S3. Inhibition of binding of human ^{125}I -Insulin to human IR-B by analogs Page S16

Figure S4. Inhibition of binding of human ^{125}I -IGF-1 to human IGF-1R by analogs Page S17

Figure S5. Dose-response curves for accelerated dissociation Page S18

Figure S6: Representative Western-blots Page S19

Figure S7: In-Cell Western Page S20

References Page S21

Supplementary Methods

Methodology for the synthesis of Fmoc- L-Lys(Pac)-OH for peptide synthesis

We have modified a previously published method (1) for preparation of Fmoc-L-Lys(Pac)-OH. The modified method is shown in the Scheme.

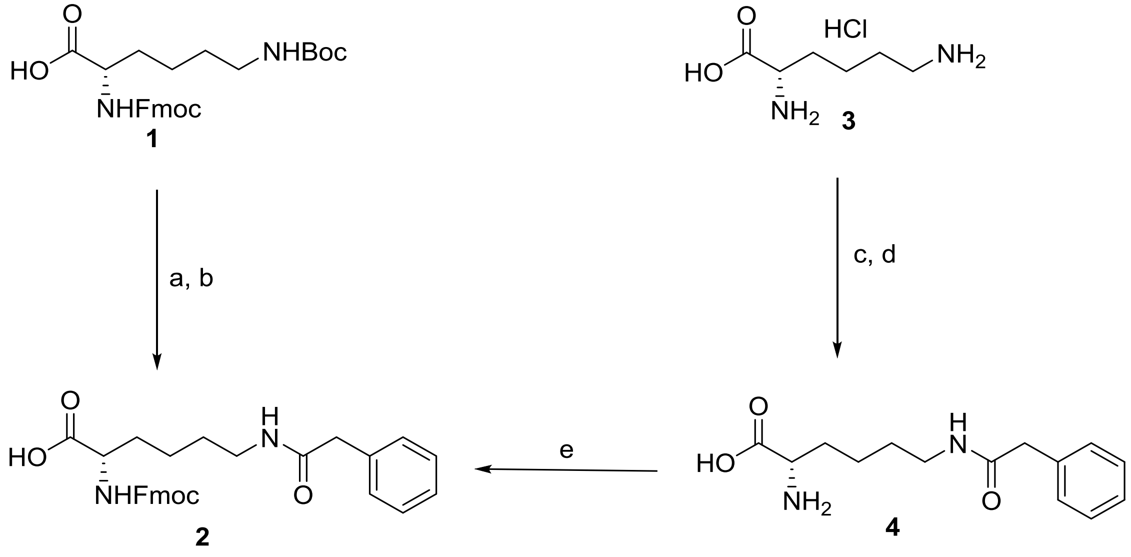

Scheme: Reagents, conditions and yields: (a) 35% HCl, water and dioxane, 1 d at rt (b) $NaHCO_3$, $PhCH_2COCl$, water and dioxane, 0°C 1 hour then rt overnight (62% over two steps); (c) $CuAc.H_2O$, NaOH, $NaHCO_3$, $PhCH_2COCl$ 0° C 1 hour then rt for 3 d; (d) 8-hydroxyquinoline, water, acetone, 4 hours at rt (60% over two steps); (e) Fmoc-OSu, $NaHCO_3$ water, dioxane, 0°C 1 hour then rt overnight (73%).

Fmoc-L-Lys(Pac)-OH (**2)**

Water (15 mL) and hydrochloric acid (11 M, 40 mL) were added to a solution of Fmoc- L-Lys(Boc)-OH **1** (26.2 g, 55.96 mmol) (2) in 150 mL of dioxane. The reaction mixture was stirred for 1 d. TLC analysis revealed complete Boc-deprotection of starting compound **1**. Then, 100 mL of water was added to the reaction mixture, followed by a careful addition of $NaHCO_3$ (56.4 g, 672 mmol, 12 equiv.) in small portions under intensive stirring. Phenylacetyl chloride (14.8 ml mL, 112 mmol, 2 equiv.) was then added slowly by a syringe and the reaction mixture was stirred overnight at rt. Then,

citric acid (64.5 g, 335.8 mmol, 6 equiv.) was added in small portions under intensive stirring and the acidified mixture (pH ~ 3) was partitioned between ethyl acetate (400 mL) and water (400 mL). The aqueous phase was washed with ethylacetate (3 x 200 mL) and combined organic layers were washed with 200 ml water, brine (3 x 200 mL), dried over Na_2SO_4, filtered and evaporated *in vacuo*. The crude product was dissolved in boiling EtOAc and purified by crystallization at -20 °C overnight. The product was filtered, washed with 100 mL of chilled (-10 °C) EtOAc and dried *in vacuo*. Alternatively, the product can be purified by flash chromatography on silica, using elution with a linear gradient of ethyl acetate-acetone-methanol-water 4 : 1: 1: 1 mixture in EtOAc containing 1 % AcOH, but only small amounts are accessible by this method due to the limited solubility of the compound.. White solid (16.9 g, 62 %), m.p. 132-134 °C. R_f = 0.55 (ethyl acetate-acetone-methanol-water 6 : 1: 1: 0.5). Calculated for $C_{29}H_{30}N_2O_5$ (268.35): 71.59 % C, 6.21 % H, 5.76 % N. Found: 71.20 % C, 6.13 % H, 5.45 % N. $[\alpha]_D^{20}$ = -10.9 (c = 0.312 ; DMF). ^{1}H NMR (600 MHz, DMSO): 1.33 (2H, m, $-CH_2-$), 1.41 (2H, m, $-CH_2-$), 1.61 (1H, m, -CHaHb-), 1.71 (1H, m, -CHaHb-), 3.04 (2H, m, $-CH_2-$), 3.39 (2H, s, $CO-CH_2-$), 3.93 (1H, ddd, *J* = 9.6, 8.0 and 4.6, -CH(CO)-N), 4.23 (1H, t, *J* = 7.1, >CH-), 4.29 (2H, d, *J* = 7.1, $-CH_2-O$), 7.20 (1H, m, ArH), 7.26 (2H, m, 2x ArH), 7.28 (2H, m, 2x ArH), 7.33 (2H, m, 2x ArH), 7.41 (2H, m, 2x ArH), 7.63 (1H, d, *J* = 8.0, -NH-CO), 7.74 (2H, m, 2x ArH), 7.89 (2H, m, 2x ArH), 12.58 (1H, br, COOH); ^{13}C NMR (150.9 MHz, DMSO): 23.32 (CH_2), 28.87 (CH_2), 30.64 (CH_2), 38.65 (CH_2), 42.65 (CH_2), 46.87 (>CH-); 53.98 (CH-N), 65.81 (CH_2-O), 120.31 (Ar=CH-), 120.33 (Ar =CH-), 125.49 (Ar =CH-), 125.51 (Ar =CH-), 126.46 (Ar =CH-), 127.27 (2x Ar =CH-), 127.85 (2x Ar =CH-), 128.37 (2x Ar=CH-), 129.13 (2x Ar=CH-), 136.76 (Ar >C=), 140.92 (Ar >C=), 140.94 (Ar >C=), 144.01 (Ar >C=), 144.06 (Ar >C=), 156.39 (N-CO-O), 170.11 (N-CO-), 174.21 (COOH). IR (KBr) ν_{max} cm^{-1} 3416 s, 3341 s (NH); 3065 m, 3031 m, 1617 s, 1478 m, 1451 s, 760 s, 740 s (rings); 1719 vs (C=O acid), 1701 (C=O carbamate), 1651 s (C=O amide); 1533 s (amide II). HRMS (ESI) calc for $C_{29}H_{30}O_5N_2Na$ $[M+Na]^+$ 509.2047, found: 509.2047.

Alternatively, L-Lys(Pac)-OH **4** (4.6 g; 17.4 mmol) was placed in a 250 ml round-bottom flask, equipped with a magnetic spin bar and suspended and sonicated in a solution of $NaHCO_3$ (2.9 g ; 34.8 mmol) in 100 ml of water. The flask was immersed in an ice-cooling bath, and Fmoc-OSu (5.9 g; 17.4 mmol) in 100 ml of dioxane was added dropwise under stirring during a period of 10 min. When the addition of Fmoc-OSu was complete, the reaction mixture (white emulsion) was allowed to react for one hour at 0 °C and then 3 days at room temperature. Then, the flask was again cooled in an ice bath and concentrated HCl was carefully added dropwise until pH ~ 1 was reached. The reaction mixture was

partitioned between 200 ml of water and 3 x 100 ml of ethyl acetate; combined organic layers were washed 1 x 50 ml water, 2 x 50 brine and dried with Na_2SO_4. The white solid was purified by crystallization from ethyl acetate. Yield 6.2 g (73 %).

L-Lys(Pac)-OH (**4**)

$CuAc_2.H_2O$ (9.98 g ; 50 mmol) in 200 ml of water was added to a stirred solution of L-Lys.HCl (18.3 g ; 100 mmol) in 100 ml of aqueous 2M NaOH. When the dark blue reaction mixture became clear, $NaHCO_3$ (17.4 g ; 200 mmol) was added, and phenyacetyl chloride (13.2 mL; 100 mmol) was added dropwise under ice-cooling and stirring. The reaction mixture was allowed to react for 1 h at 0 °C, then at rt overnight. The precipitate was filtered off in a Büchner funnel, washed with 400 mL of water, 200 mL of acetone and dried under deep vacuum. Next, finely grounded $Lys_2(Pac)_2Cu$ (23.3 g ; 39.3 mmol) was suspended in a mixture of 50 mL water and 50 mL acetone. Then, 8-hydroxyquinol (14.8 g ; 102.18 mmol) and 400 mL water were added to a vigorously stirred slurry. After 4 hours, a green precipitate was filtered off in a Büchner funnel and washed with 200 mL of water. The filtrate was extracted 3 times with 200 mL of ethyl acetate in a separatory funnel. The aqueous layer was separated and evaporated under reduced pressure. Crystals of the product were obtained by a crystallization from water. Yield 16 g (60 %). White solid (zwitterion), m.p. 220-222 °C. $[\alpha]_D^{20}$ = +12.3 (c = 0.332 ; 1M HCl). ^{1}H NMR (600 MHz, D_2O+NaOD): 1.30 (2H, m, -CH_2-), 1.50 (3H, m, -CH_2- and –C<u>Ha</u>Hb-), 1.59 (1H, m, -CHa<u>Hb</u>-), 3.18 (3H, t, J ~ 9.2, -CH_2-N + -CH(CO)-N), 3.56 (2H, s, CO-CH_2-), 7.34 (3H, m, Ar-H), 7.42 (2H, m, Ar-H); ^{13}C NMR (150.9 MHz, D_2O+NaOD): 25.14 (CH_2), 31.03 (CH_2), 37.10 (CH_2), 42.06 (CH_2-N), 58.60 (CH-N), 130.06 (Ar =CH-), 131.72 (2x Ar =CH-), 131. 82 (2x Ar =CH-), 138.05 (Ar >C=), 176.91 (N-CO), 186.00 (COOH). IR (KBr) ν_{max} cm^{-1} 3431 m, 3315 m (NH); 3085 w, 3065 w, 3031 m, 1603 m, 1582 s, 1478 m, 1454 m, 695 m (rings); 1719 vs (C=O acid), 1664 vs (C=O acid), 1639 vs (C=O amide); 1537 m (amide II). HRMS (ESI) calc for $C_{14}H_{19}O_3N_2$ $[M-H]^+$ 263.1401, found: 263.1402.

Preparation of ^{125}I-[D-HisB24, GlyB31, TyrB32]-insulin

An Eppendorf PP tube (1.5 mL), pre-coated *in house* with 10 nmoles of IODO-GEN™ (Pierce), was rinsed with 200 µL of PBS (0.1 M phosphate buffer pH 7.6 and 0.15 M NaCl). Fresh PBS (200 µL), sodium iodide (1 µL Na[^{125}I], 0.1 mCi, product code: I-RB-41, IZOTOP, Hungary) and aqueous solution of the insulin analog 100 µL (1 µg/µL) were mixed in the reaction tube. The tube was vigorously

shaken for 10 min at room temperature and then 200 μL of 20 mM HEPES buffer (pH 7.3) containing BSA (1 mg/mL) was added. BSA (void of interfering IGF-like binding proteins, product code: A6003, Sigma) was used to suppress the stickiness of the peptide to the reaction tube walls.

The mixture was directly injected into the HPLC system (Analytical-preparative radio-HPLC, pump Waters 600, UV detector Waters 2487, radio chromatogram detector Ramona (Raytest, Germany). The desired mono-iodinated product was separated from the unmodified and over-iodinated analog on Nucleosil 120 C18 column (5μ, 250 × 4.0 mm, Watrex) at 25 °C. The mobile phases were: A, 0.1 % (v/v) TFA and B, 0.1 % (v/v) TFA in 100 % acetonitrile. The flow rate was 1 mL/min. The gradient started at 20 % mobile phase B, remained constant for the first 5 min, followed by a linear increase to 32.5 % mobile phase B over 70 min. Fractions were collected to tubes with 200 μL of BSA cocktail (12.5 mM sodium phosphate pH 7.4, 50 mM NaCl, 0.5 M glycine and 0.06 % BSA). The isolated fractions containing ^{125}I-[D-HisB24, GlyB31, TyrB32]-insulin were transferred to LoBind® Eppendorf tubes and evaporated to dryness on CentriVap at room temperature for 4 hours. The non-iodinated analog and three fractions of iodinated product were separated. Radioactivity was measured using the Gamma Counter Wizard 1470 (Perkin Elmer).

Retention times and identities of the separated products were assessed, based on previous mass spectrometry analysis of a non-radioactive iodination experiment performed with [^{127}I]-NaI. The retention time of the unmodified analog was 38 min. The desired mono-iodinated product at Tyr32 had a retention time of 44 min. We finally obtained 15 μCi of mono-iodinated ^{125}I-[D-HisB24, GlyB31, TyrB32]-insulin at Tyr32 (15.0 % RCY, >99 % RCP). Two other fractions were separated. These fractions were double or triple iodinated. The fraction with the retention time of 42 min contained the analog with prevailing iodination at position TyrB26, and the fraction at 47 min contained the analog iodinated at all three positions (TyrB16, TyrB26 and TyrB32). No iodination was detected on the A chain.

Mass spectrometry analysis of iodinated fractions was performed using UltrafleXtreme™ MALDI-TOF/TOF (Bruker Daltonics). At first, the extent of iodination was determined (analysis conditions: sample diluted in 50 % acetonitrile with 0.1 % TFA was applied on DHB matrix and analyzed in linear mode, instrumental setting tuned to 5 - 20 kDa). Then, cysteines were reduced and alkylated, using iodoacetamide to distinguish among iodination on A chain or B chain (analysis conditions: sample diluted in 50 % acetonitrile with 0.1 % TFA was applied on DHB matrix and analyzed in reflectron mode, instrumental setting tuned to 2 - 6 kDa). The position of Tyr residue

modified by iodine was revealed after digestion with trypsine through LC-MS/MS peptide analysis on ULTIMATE 3000 RSLCnano (Dionex, Thermo Scientific) coupled to TripleTOF™ 5600 (SCIEX). The extent of iodination in a specific position in the three fractions containing iodinated Tyr residues was approximated by comparing the area of peaks corresponding to peptides containing TyrB16, TyrB26 and TyrB32 and their iodinated counterparts.

Receptor-binding Studies

Human IM-9 lymphocytes (containing IR-A) and IGF-1R null mouse embryonic fibroblasts, stably transfected with either human IR-B or human IGF-1R, were employed for whole cell receptor-binding assay as described previously (3-5).

IM-9 cells 2 x 10^6/mL were incubated with (0.01 nM - 10 μM) dilutions of ligands and human mono-^{125}I-Insulin (Perkin Elmer, 20,000 cpm, about 0.01 nM) for 2.5 h at 15°C in 500 μL of HEPES binding buffer (100 mM HEPES, 100 mM NaCl, 5 mM KCl, 1.3 mM $MgSO_4$, 1 mM EDTA, 10 mM glucose, 15 mM NaOAc, 1 % w/v BSA, pH 7.6). After incubation 2 x 200 μL were centrifuged at 15,000 x g for 10 min in microfuge tubes containing 200 μL of pre-cooled HEPES binding buffer. Radioactive pellets were counted using the Gamma Counter Wizard 1470 (Perkin Elmer). Binding data were analyzed by Excel software using a method of non-linear regression and a one-site fitting program developed in the laboratory of Dr. Pierre De Meyts (A.V. Groth and R.M. Shymko, Hagedorn Research Institute, Denmark, a kind gift of Pierre De Meyts). The dissociation constant of human ^{125}I-insulin was set up to 0.3 nM. Receptor binding assays were performed under conditions excluding the depletion of free ligand. The software used for the analysis of binding data developed in the laboratory of Dr. Pierre De Meyts takes the potential ligand depletion into account. The binding curve of each analog was determined in duplicate and the final dissociation constant (K_d) was calculated from at least three (n≥3) binding curves. Significance of the changes in binding affinities in relation to insulin or IGF-1 was calculated using the two-tailed *t* test.

IGF-1R null mouse embryonic fibroblasts, stably transfected with either human IR-B or human IGF-1R were seeded in 24-well plates (Schoeller) (12x10^3 cells per well) in 300 μL of DMEM media and incubated for 24 hours. Cells were afterwards starved for 4 hours in serum-free media. The cells were incubated with (0.01 nM - 10 μM) dilutions of ligands and human mono-^{125}I-Insulin (IR-B) or mono-^{125}I-IGF-1 (IGF-1R) (Perkin Elmer, 20,000 cpm, about 0.01 nM) for 16 h at 5°C in 250 μL of

HEPES binding buffer. The use of bovine serum albumin in the binding buffer (e.g. Sigma-Aldrich A6003) void of 'IGF-binding-like' proteins, which interfere with the binding assay of IGF-1 is essential. Following the incubation, the cells were washed twice with ice-cold binding buffer and solubilized with 0.1 M NaOH. Cell-associated radioactivity was counted using the Gamma Counter Wizard 1470 (Perkin Elmer). Binding data were analyzed by and the dissociation constant (K_d) was determined with GraphPad Prism 5 using a non-linear regression, a one-site fitting program and taking into account potential depletion of free ligand. The dissociation constant of human ^{125}I-insulin was set up to 0.3 nM. The dissociation constant of human ^{125}I-IGF-1 was set up to 0.2 nM. Each binding curve was determined in duplicate and the final dissociation constant (K_d) was calculated from at least three (n≥3) binding curves.

For dose-response curves for accelerated dissociation the cells (3 x 10^7/mL) in HEPES binding buffer were preincubated with the radio-labeled ligand (about 2500 Ci/mmol, 150,000 cpm/mL, about 0.03 nM) for 2.5 hours at 15°C. After the preincubation, the suspension was centrifuged at 1000 x g, 4°C for 5 min and the supernatant containing the unbound radioactivity was discarded. The pellet was resuspended in the initial volume of buffer. Duplicate aliquots were diluted 40 times and incubated in increasing amounts of cold ligand (0.001 nM – 10 μM) for 30 min at 15°C. A duplicate aliquot where no cold ligand was added was incubated at the same conditions. After the incubation the aliquots were centrifuged at 600 x g, 4°C for 5 min, supernatant was discarded and the remaining radioactivity was counted using the Gamma Counter Wizard 1470 (Perkin Elmer). Non-specific binding was substracted from the obtained values. IM-9 cells were used for IR-A measurements and CHO-R+ cells were used for IGF-1R. To fit the curves we used functions predefined in GraphPad Prism 5 software. IR-A curves were fitted using non-linear regression, dose-response special for bell-shaped. IGF-1R curves were fitted using non-linear regression, competitive binding fitting log IC50.

For association kinetics, the cells (10^7/mL) in HEPES binding buffer were incubated with the radio-labeled ligand (about 2500 Ci/mmol, 150,000 cpm/mL, about 0.03 nM) at 15°C. At specific time points, duplicate aliquots of 200 μL were centrifuged at 15,000 x g, 4°C for 10 minutes in microfuge tubes containing 200 μL of pre-cooled HEPES binding buffer. The supernatant was discarded and the remaining radioactivity in the pellet was counted using the Gamma Counter Wizard 1470 (Perkin Elmer).). Non-specific binding was substracted from the obtained values. Duplicate aliquots of 200 μL of non-centrifuged cells were counted as total. IM-9 and CHO-R+ cells were used as described for dose-

response curves for accelerated dissociation. To fit the curves we used a function for association kinetics – one concentration of hot predefined in the GraphPad Prism 5 software.

For dissociation kinetics, the cells ($3x10^7$/mL) in HEPES binding buffer were preincubated with the radio-labeled ligand (about 2500 Ci/mmol, 150,000 cpm/mL, about 0.03 nM) at 15°C for 2.5 hours in the case of insulin and analog binding to IR-A. In the case of IGF-1 binding to IR-A, the preincubation lasted 4 hours. The period was 2.5 hours for analog and IGF-1 binding to IGF-1R, and 7 hours in the case of insulin binding to IGF-1R. The preincubation duration was estimated based on association kinetics (time after reaching the steady state). After the preincubation, the cell suspension was centrifuged at 1000 x g. 4°C for 5 min. The supernatant was discarded and the pellet resuspended in the initial volume of buffer. Duplicate aliquots were diluted 40 times in presence or absence of cold ligand and incubated at 15°C. The concentrations of cold ligand used were 170 nM for all the experiments, except for insulin on IGF-1R, where a 17 μM solution of insulin was used. At specific time points, duplicate aliquots were centrifuged at 600 x g, 4°C for 5 min. The supernatant was discarded and the remaining radioactivity in the pellet was counted using the Gamma Counter Wizard 1470 (Perkin Elmer). IM-9 and CHO-R+ cells were used as described for dose-response curves for accelerated dissociation. To fit the curves we used a model for dissociation – one phase exponencial decay predefined in the GraphPad Prism 5 software.

Receptor Phosphorylation Assay

Cell stimulation and detection of receptor phosphorylation were performed as described previously (5). Shortly, mouse fibroblasts (IR-A, IR-B and R^{+39}) were seeded in 24-well plates (Schoeller) ($4x10^4$ cells per well) in 300 μL of DMEM media and incubated for 24 hours. Cells were afterwards starved for 4 hours in serum-free media. The cells were stimulated with 10 nM concentrations of the ligands for 10 min. The reaction was terminated by washing monolayers in 1 mL ice-cold 0.9% NaCl followed by snap freezing in liquid nitrogen. The cells in individual wells were lysed in 50 μL of lysis buffer (62.5 mM Tris/Cl pH 6.8, 2% w/v SDS, 10% v/v glycerol, 0.01% w/v bromfenol blue, 100 mM DTT, 50 mM NaF, 1 mM Na_3VO_4 and 0.5% v/v protease inhibitor coctail (Sigma; P8340).

Proteins were routinely analyzed using immunoblotting and horseradish peroxidase-labeled secondary antibodies (Sigma-Aldrich). Cell extracts (10 μL containing 10 ± 0.8 μg proteins) were separated on 10 % SDS polyacrylamide gels and electroblotted to PVDF membrane. The membranes were probed with

anti-phospho-IGF-1Rβ (Tyr1135/1136)/IRβ (Tyr1150/1151) (Cell Signaling Technology) or with anti-actin (20-33) (Sigma-Aldrich). The blots were developed using the SuperSignal West Femto maximum sensitivity substrate (Pierce) and analyzed using the ChemiDoc MP Imaging System (Bio-Rad). Each experiment was repeated at least four times. Each blot contained an internal standard sample stimulated with 10 nM insulin (IR-A and IR-B) or with 10 nM IGF-1 (IGF-1R) which was used to calculate the relative intensities. The data were expressed as the contribution of phosphorylation relative to the human insulin (IR-A, IR-B) or IGF-1 (IGF-1R) signal. Mean ± S.D. ($n \geq 4$) values were calculated. The significance of the changes in stimulation of phosphorylation in relation to insulin was calculated, using one-way analysis of variance (ANOVA) with Dunnett's test comparing all analogs versus control i.e. insulin.

Ligand-dose response IGF-1R autophosphorylation levels for selected analogs were determined, using In-Cell Western assay adapted for chemiluminiscence as described in (6). The IGF-1R cells were plated at 20,000 cells/well in white 96-well Brand *plates* cell grade (Brand GMBH, Germany) and incubated for 24 hours. Cells were afterwards starved for 4 hours in serum-free media and stimulated with dilutions of ligands (0-100 nM) for 20 min. After the incubation, the medium was discarded and the cells were fixed in 3.75 % v/v freshly prepared formaldehyde for 20 min. Cells were permeabilized with 0.1 % v/v Triton-X-100 in PBS for 5 min and blocked with 5% w/v BSA in T-TBS for 1 hour. Plates were incubated with anti-phospho-IGF-1Rβ (Tyr1135/1136)/IRβ (Tyr1150/1151) overnight at 4°C. Then plates were thoroughly washed with TBS and incubated with peroxidase-labeled anti-rabbit secondary antibody (Sigma) for 1 hour at room temperature, and washed again. SuperSignal West Femto maximum sensitivity substrate was added to each well and chemiluminiscence was detected using the ChemiDoc MP Imaging System after 5 min. The ligands were tested in triplicates on each plate and intensities after subtraction of background (wells with no ligand) were related to the average intensity detected in wells stimulated with 10 nM IGF-1 on the particular plate (i.e. average intensity detected in 3 wells per plate stimulated with 10 nM IGF-1). Experiments were repeated four times. Log(agonist) vs. response curve fitting of data was carried out with GraphPad Prism5 software.

Supplementary Tables

Table S1: Source data for calculation of kinetics coefficient ratios.

Receptor type	Parameter	Insulin	IGF-1	[D-HisB24,GlyB31,TyrB32]-insulin
IR-A	[a] K_d ± S.D. [nM] (n)	0.36 ± 0.14[#](5)<br>0.45 ± 0.11[$](6)	31.51 ± 5.63[#](4)	0.18 ± 0.02[$](3)
	[b] (d1) dissociation rate at maximum acceleration ± S.D. [min^{-1}]	0.1451 ± 0.0093	0.0227 ± 0.0044	0.0432 ± 0.0032
	[c] dissociation rate (measured) ± S.D. [min^{-1}]	0.0758 ± 0.0105	0.0006 ± 0.0004	0.0017 ± 0.0002
	[c] dissociation rate (derived) [min^{-1}]	0.0124	0.0124/6.39 = 0.0019	0.0124/3.36 = 0.0037
	[d] (a) association rate ± S.D. [$M^{-1}min^{-1}$] using (measured) dissociation rate	(3.09 ± 0.08)E9	(2.73 ± 1.93)E9	(3.52 ± 0.82)E9
	[d] association rate ± S.D. [$M^{-1}min^{-1}$] using (derived) dissociation rate	(4.32 ± 0.08)E9	(2.68 ± 1.93)E9	(3.47 ± 0.82)E9
IGF-1R	[a] K_d ± S.D. [nM] (n)	292 ± 54.3[€](3)	0.24 ± 0.10[€](5)<br>0.11 ± 0.05[$](5)	0.89 ± 0.20[$] (6)
	[b] (d1) dissociation rate at maximum acceleration ± S.D. [min^{-1}]	0.0069 ± 0.0004	0.0214 ± 0.0012	0.0203 ± 0.0038
	[c] dissociation rate (measured) ± S.D. [min^{-1}]	0.0026 ± 0.0010	0.0032 ± 0.0029	0.0064 ± 0.0035
	[c] dissociation rate (derived) [min^{-1}]	0.0048/3.1 = 0.0015	0.0048	0.0048/1.05 = 0.0046
	[d] (a) association rate ± S.D. [$M^{-1}min^{-1}$] using (measured) dissociation rate	(0.32 ± 0.11)E9	(31.75 ± 4.28)E9	(4.70 ± 2.38)E9
	[d] association rate ± S.D. [$M^{-1}min^{-1}$] using (derived) dissociation rate	(0.35 ± 0.11)E9	(30.65 ± 2.90)E9	(4.76 ± 2.39)E9

[a] Dissociation constants K_d are the same as in Table 1.

[b] Dissociation rate at maximal acceleration was measured as the rate of dissociation of ^{125}I-labeled ligand in the presence of cold ligand. Ratios of dissociation factors for site 1 (d1) were calculated from these values.

[c] Dissociation rate was measured as the rate of dissociation of ^{125}I-labeled ligand without the presence of cold ligand (measured), or it was derived from a constant calculated for the first ligand dissociation in the model for negative cooperativity (derived) (7). The (derived) constants were adjusted to the respective analog according to the found (d1) ratios.

[d] Association rates were obtained after fitting the experimental data to association kinetics in GraphPad Prism 5 software, using both the (measured) and the (derived) values of dissociation constants [c].

Table S2: Chemical shift differences of backbone HN and Hα protons of [D-HisB24, GlyB31, TyrB32] -insulin and [D-HisB24]-insulin (8) at 25°C and pH 8.0*.

Residue		ΔHN	ΔHα	Residue		ΔHN	ΔHα
A1	Gly			B5	His		
A2	Ile			B6	Leu		
A3	Val	0.012	-0.027	B7	Cys	0.006	0.035
A4	Glu	0.018	-0.009	B8	Gly		
A5	Gln			B9	Ser		
A6	Cys		-0.001	B10	His		0.007
A7	Cys		0.021	B11	Leu		-0.005
A8	Thr		-0.018	B12	Val		-0.011
A9	Ser		0.019	B13	Glu	0.015	0.004
A10	Ile	0.008	0.013	B14	Ala	0.004	-0.003
A11	Cys			B15	Leu	0.010	0.003
A12	Ser	0.051	0.005	B16	Tyr	0.012	-0.008
A13	Leu		-0.012	B17	Leu	0.031	-0.020
A14	Tyr	0.014	0.002	B18	Val	0.020	-0.004
A15	Gln	-0.002	-0.002	B19	Cys	0.037	-0.028
A16	Leu	0.019	-0.004	B20	Gly	-0.009	-0.003
A17	Glu	0.002	0.003	B21	Glu		-0.002
A18	Asn	-0.008	0.005	B22	Arg	0.000	-0.005
A19	Tyr	0.008	0.003	B23	Gly	0.001	0.002
A20	Cys	-0.003		B24	D-His		
A21	Asn	0.016	-0.004	B25	Phe		0.001
				B26	Tyr	0.021	-0.007
B1	Phe		0.031	B27	Thr	0.005	0.001
B2	Val		0.008	B28	Pro		-0.004
B3	Asn		0.007	B29	Lys	-0.025	-0.033
B4	Gln		0.018	B30	Thr		0.174

* Due to slightly different referencing of our NMR shifts and shifts from (8) data were re-referenced by 0.038 ppm for the purpose of this comparison only.

Table S3: Proton NMR data of [D-HisB24, GlyB31, TyrB32]-insulin at 25°C and pH 8.0.

Residue		HN	Hα	Hβ	Hγ	Hδ	Hε
A1	Gly			-	-	-	-
A2	Ile						-
A3	Val	8.21	3.67	1.95	0.92, 0.88	-	-
A4	Glu	8.40	4.16	2.03, 1.94	2.43, 2.25	-	-
A5	Gln						-
A6	Cys		5.09	3.41, 2.89	-	-	-
A7	Cys		4.88	3.71, 3.30	-	-	-
A8	Thr		4.16	4.43	1.24	-	-
A9	Ser		4.68	3.94, 3.84	-	-	-
A10	Ile	7.87	4.19	1.60	0.67, 1.12	0.52	-
A11	Cys				-	-	-
A12	Ser	8.63	4.60	4.16, 4.00	-	-	-
A13	Leu		3.95	1.56	1.50	0.82, 0.88	-
A14	Tyr	7.72	4.35	3.00	-	7.11	6.85
A15	Gln	7.57	3.98	2.39, 2.23	2.34, 2.05	-	
A16	Leu	7.95	4.18	1.96, 1.49	1.78	0.82	-
A17	Glu	8.19	4.10	2.04, 1.94	2.14, 2.41	-	-
A18	Asn	7.45	4.49	2.55	-	6.60, 7.22	-
A19	Tyr	7.97	4.54	3.32, 3.00	-	7.28	6.78
A20	Cys	7.57	4.78	3.31, 2.89	-	-	-
A21	Asn	7.97	4.49	2.77, 2.61	-	7.34, 6.72	-
B1	Phe		3.83	3.02, 2.97	-	7.16	7.28
B2	Val		4.01	1.92	0.81	-	-
B3	Asn		4.56	2.75, 2.81	-		-
B4	Gln		4.46	2.05, 1.93	2.21, 2.13	-	7.40, 6.81
B5	His				-		
B6	Leu						-
B7	Cys	8.49	4.96		-	-	-
B8	Gly			-	-	-	-
B9	Ser				-	-	-
B10	His		4.49	3.33, 3.21	-		
B11	Leu		4.06		1.42	0.76, 0.89	-
B12	Val		3.41	2.11	0.97, 0.94	-	-
B13	Glu	7.99	4.06	2.10	2.29, 2.43	-	-
B14	Ala	7.76	4.09	1.42	-	-	-
B15	Leu	8.21	3.93	1.56	1.56	0.72, 0.58	-
B16	Tyr	8.05	4.20	3.10	-	7.09	6.75
B17	Leu	7.67	4.12				-
B18	Val	8.32	3.85	2.07	0.90, 1.01	-	-
B19	Cys	8.73	4.78	3.3, 2.84	-	-	-
B20	Gly	7.85	3.99	-	-	-	-
B21	Glu		4.27	2.14, 1.99	2.32, 2.27	-	-
B22	Arg	8.03	4.34	2.05, 1.84	1.71, 1.68	3.20	-
B23	Gly	8.14	3.88	-	-	-	-
B24	D-His				-		
B25	Phe		4.68	3.02, 2.88	-	7.04	7.16
B26	Tyr	8.29	4.60	2.95	-	7.06	6.78
B27	Thr	7.97	4.49	4.03	1.18	-	-
B28	Pro	-	4.36	2.28, 1.97	1.90, 1.90	3.61	-
B29	Lys	8.42	4.36	1.85, 1.77	1.43, 1.48	1.66	2.96
B30	Thr		4.34	4.18	1.18	-	-
B31	Gly	-	3.84, 3.94	-	-	-	-
B32	Tyr	7.68	4.41	-	-	7.09	6.82

Supplementary Figures

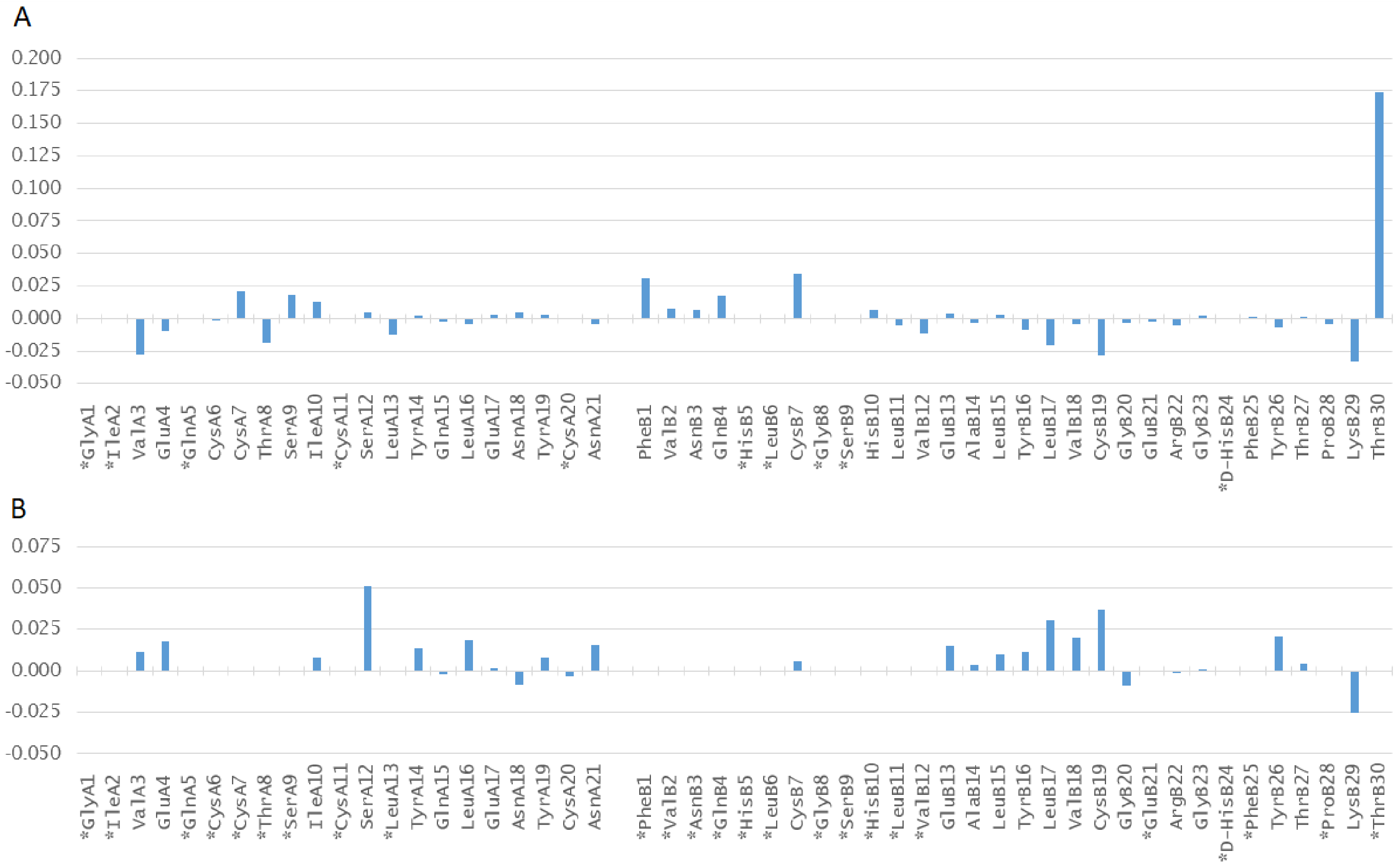

Figure S1: **Graphical presentation of chemical shift differences** of Hα (**A**) and backbone HN (**B**) protons of [D-HisB24, GlyB31, TyrB32]-insulin and [D-HisB24]-insulin at 25°C and pH 8.0. Data for residues marked by asterisk (*) are not available.

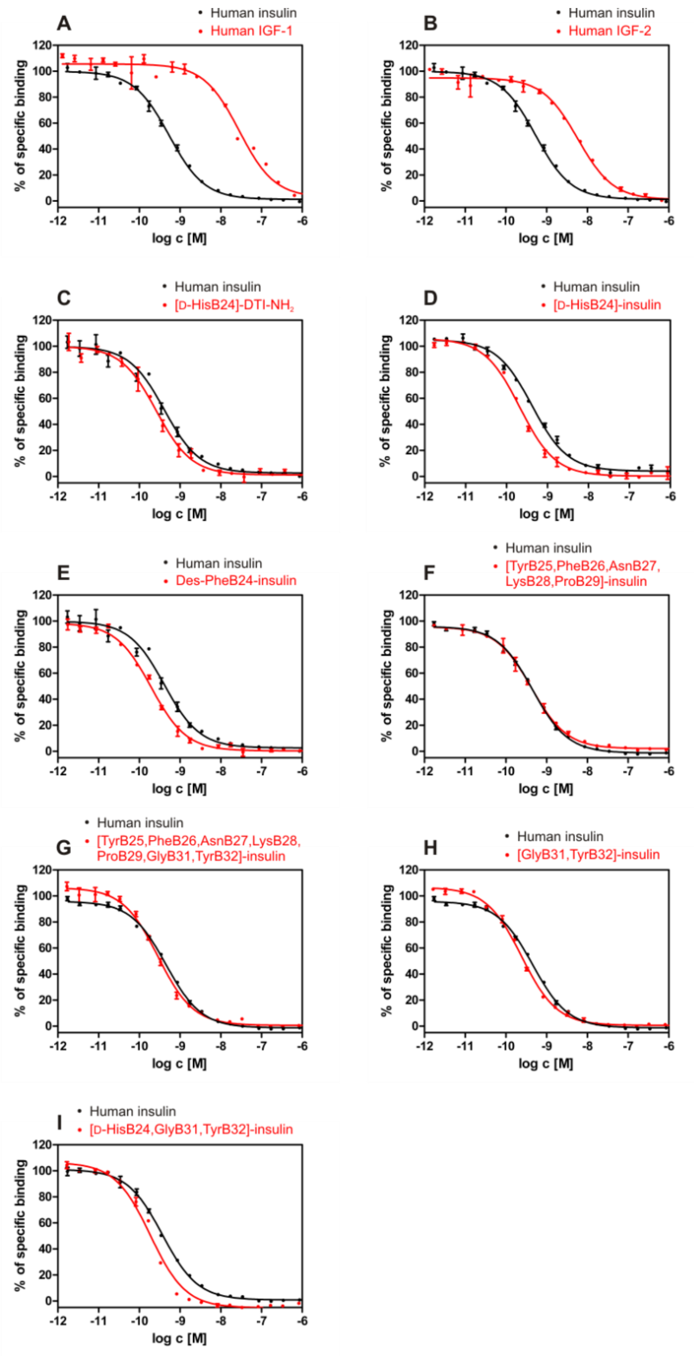

Figure S2. Inhibition of binding of human ^{125}I-Insulin to human IR-A by analogs in IM-9 cells by (**A**) human IGF-1, (**B)** human IGF-2, (**C)** [D-HisB24]-DTI-NH_2, (**D**) [D-HisB24]-insulin, (**E**) des-PheB24-insulin, (**F**) [TyrB25, PheB26, AsnB27, LysB28, ProB29]-insulin, (**G**) [TyrB25, PheB26, AsnB27, LysB28, ProB29, GlyB31, TyrB32]-insulin, (**H**) [GlyB31, TyrB32]-insulin, (**I**) [D-HisB24, GlyB31, TyrB32]-insulin and human insulin (in all panels). The representative binding curves are shown.

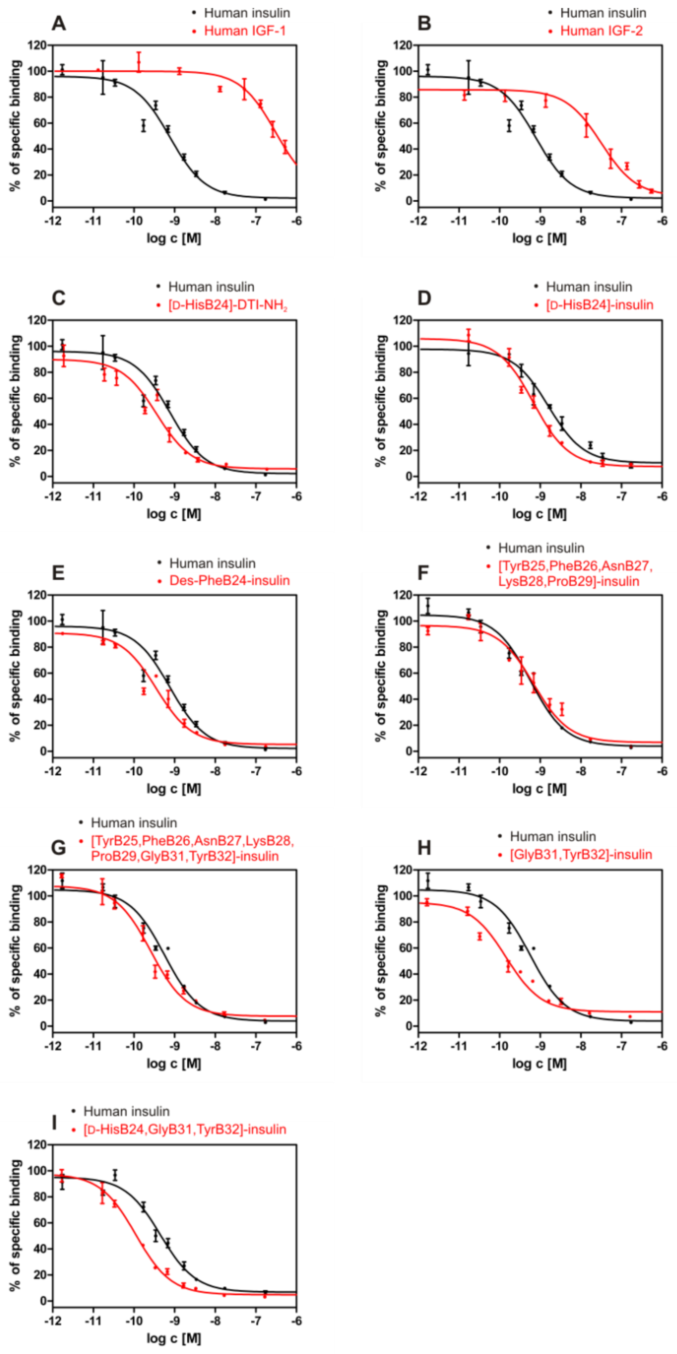

Figure S3. Inhibition of binding of human ^{125}I-Insulin to human IR-B by analogs in mouse embryonic fibroblast by (**A**) human IGF-1, (**B)** human IGF-2, (**C)** [D-HisB24]-DTI-NH_2, (**D**) [D-HisB24]-insulin, (**E**) des-PheB24-insulin, (**F**) [TyrB25, PheB26, AsnB27, LysB28, ProB29]-insulin, (**G**) [TyrB25, PheB26, AsnB27, LysB28, ProB29, GlyB31, TyrB32]-insulin, (**H**) [GlyB31, TyrB32]-insulin, (**I**) [D-HisB24, GlyB31, TyrB32]-insulin and human insulin (in all panels). The representative binding curves are shown.

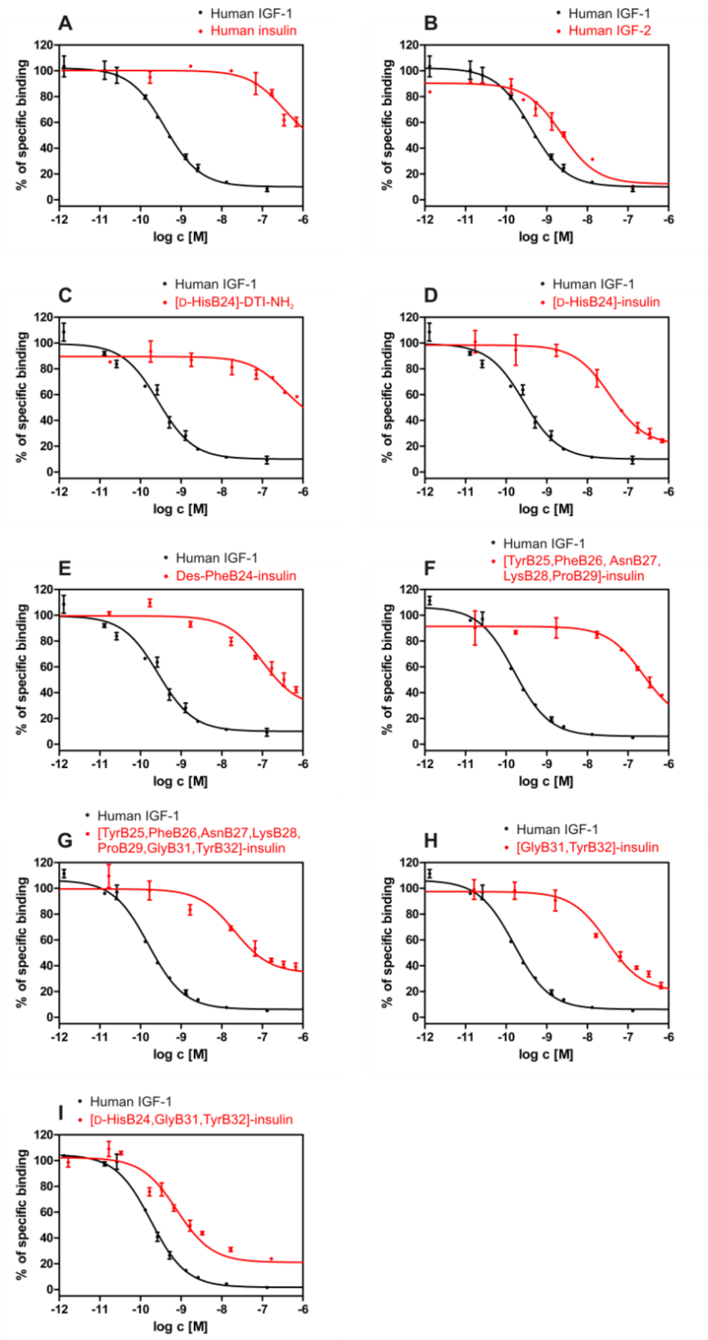

Figure S4. Inhibition of binding of human ^{125}I-IGF-1 to human IGF-1R by analogs in mouse embryonic fibroblast by (**A**) human insulin, (**B)** human IGF-2, (**C)** [D-HisB24]-DTI-NH_2, (**D**) [D-HisB24]-insulin, (**E**) des-PheB24-insulin, (**F**) [TyrB25, PheB26, AsnB27, LysB28, ProB29]-insulin, (**G**) [TyrB25, PheB26, AsnB27, LysB28, ProB29, GlyB31, TyrB32]-insulin, (**H**) [GlyB31, TyrB32]-insulin, (**I**) [D-HisB24, GlyB31, TyrB32]-insulin and human insulin (in all panels). The representative binding curves are shown.

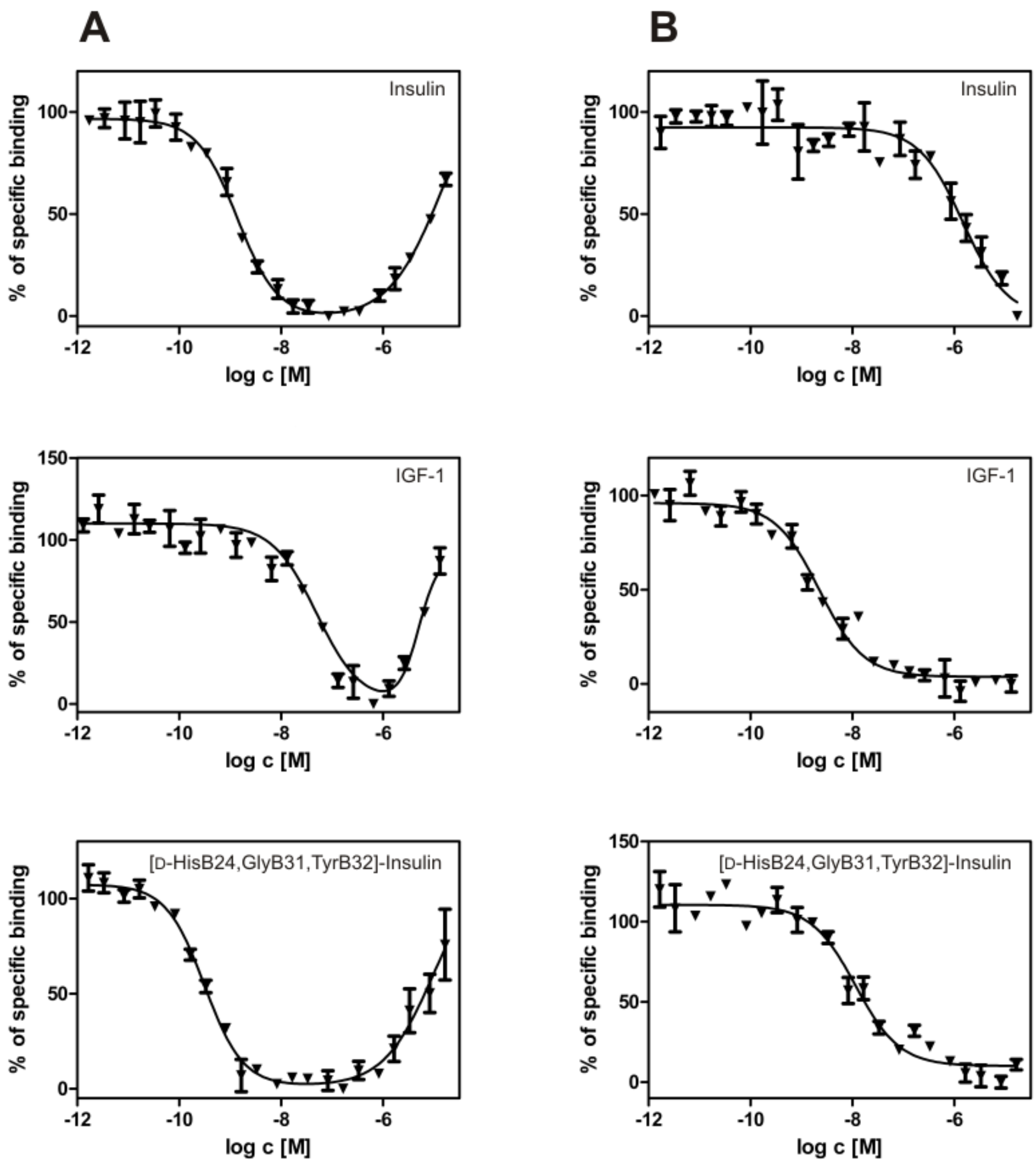

Figure S5. Dose-response curves for accelerated dissociation. A: Dissociation of prebound ^{125}I-Insulin in the presence of increasing concentration of human insulin, IGF-1 or [D-HisB24, GlyB31, TyrB32]-insulin from IR-A. The experiments were performed on human IM-9 lymphocytes. B: Dissociation of prebound ^{125}I-IGF-1 in the presence of increasing concentration of human insulin, IGF-1 or [D-HisB24, GlyB31, TyrB32]-insulin from IGF-1R. The experiments were performed on CHO-K1 cell line stably transfected with IGF-1R. The dissociation is illustrated as the percentage of radioactivity remaining after dissociation for 30 min. Bound radioactivity at zero concentration of cold ligand was set as 100 %. Representative curves are shown.

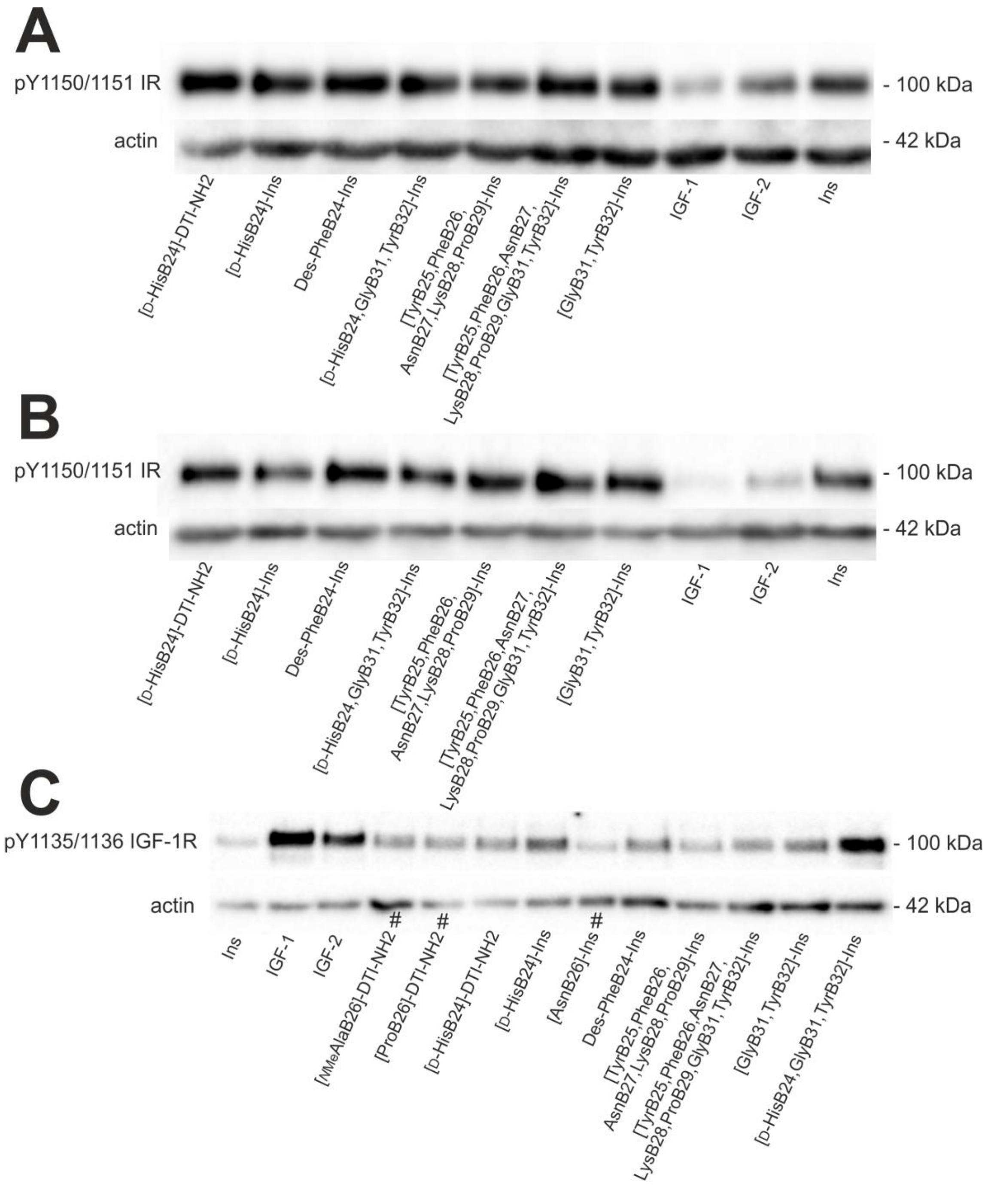

Figure S6: Representative western blots for relative abilities of human insulin, IGF-1, IGF-2 and insulin analogs to stimulate receptor phosphorylation. A) IR-A transfected fibroblasts. B) IR-B transfected fibroblasts. C) IGF-1R transfected fibroblasts. Cells were stimulated with 10 nM ligands for 10 min. Membranes were cut at 75 kDa and 50 kDa standards and respective parts were developed with anti-phospho-IGF-1Rβ (Tyr1135/1136)/IRβ (Tyr1150/1151) antibody (Mr above 75kDa) and with anti-actin antibody (Mr bellow 50 kDa). # IGF-1R transfected cells were also stimulated with marked analogs [*N*MeAlaB26]-DTI-NH_2, [ProB26]-DTI-NH_2 (9) and [AsnB26]-insulin (4) that were not discussed in the manuscript.

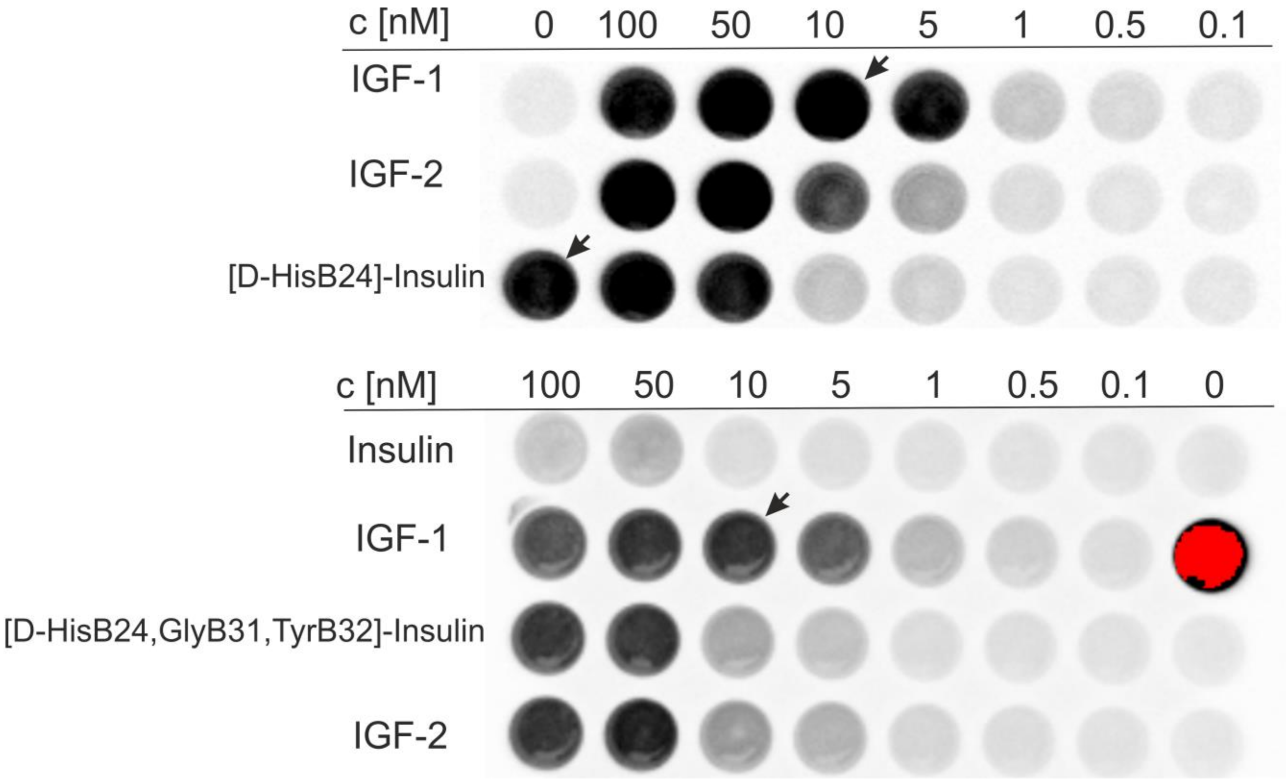

Figure S7. In-Cell Western. Stimulation of phosphorylation of receptors in IGF-1R transfected fibroblasts with 0.1-100 nM concentration range of the ligands for 20 min. The cells in the wells of 96-well plate were fixed with formaldehyde and the extent of phosphorylation was detected by anti-phospho-IGF-1Rβ (Tyr1135/1136)/IRβ (Tyr1150/1151) antibody. Signal was developed using the SuperSignal West Femto maximum sensitivity substrate (Pierce) and acquired using the ChemiDoc MP Imaging System (Bio-Rad). Two representative experiments are shown. Anti-IGF-1R β-subunit (C-20) antibody (Santa Cruz Biotechnology) was used in the well filled with red coloring showing total receptor content. Arrows indicate the wells treated with 10 nM IGF-1 used to calculate relative intensity.

References

1. Záková, L., Zyka, D., Jezek, J., Hanclová, I., Sanda, M., Brzozowski, A. M., and Jirácek, J. (2007) The use of Fmoc-Lys(Pac)-OH and penicillin G acylase in the preparation of novel semisynthetic insulin analogs. *J Pept Sci* 13, 334-341
2. Pícha, J., Buděšínský, M., Macháčková, K., Collinsová, M., and Jiráček, J. (2017) Optimized syntheses of Fmoc azido amino acids for the preparation of azidopeptides. *J Pept Sci* 23, 202-214
3. Morcavallo, A., Genua, M., Palummo, A., Kletvikova, E., Jiracek, J., Brzozowski, A. M., Iozzo, R. V., Belfiore, A., and Morrione, A. (2012) Insulin and insulin-like growth factor II differentially regulate endocytic sorting and stability of insulin receptor isoform A. *J Biol Chem* 287, 11422-11436
4. Záková, L., Kletvíková, E., Lepšík, M., Collinsová, M., Watson, C. J., Turkenburg, J. P., Jiráček, J., and Brzozowski, A. M. (2014) Human insulin analogues modified at the B26 site reveal a hormone conformation that is undetected in the receptor complex. *Acta Crystallogr D Biol Crystallogr* 70, 2765-2774
5. Křížková, K., Chrudinová, M., Povalová, A., Selicharová, I., Collinsová, M., Vaněk, V., Brzozowski, A. M., Jiráček, J., and Žáková, L. (2016) Insulin-Insulin-like Growth Factors Hybrids as Molecular Probes of Hormone:Receptor Binding Specificity. *Biochemistry* 55, 2903-2913
6. Macháčková, K., Collinsová, M., Chrudinová, M., Selicharová, I., Pícha, J., Buděšínský, M., Vaněk, V., Žáková, L., Brzozowski, A. M., and Jiráček, J. (2017) Insulin-like Growth Factor 1 Analogs Clicked in the C Domain: Chemical Synthesis and Biological Activities. *J Med Chem* 60, 10105-10117
7. Xu, Y., Kong, G. K., Menting, J. G., Margetts, M. B., Delaine, C. A., Jenkin, L. M., Kiselyov, V. V., De Meyts, P., Forbes, B. E., and Lawrence, M. C. (2018) How ligand binds to the type 1 insulin-like growth factor receptor. *Nat Commun* 9, 821
8. Žáková, L., Kletvíková, E., Veverka, V., Lepsík, M., Watson, C. J., Turkenburg, J. P., Jirácek, J., and Brzozowski, A. M. (2013) Structural integrity of the B24 site in human insulin is important for hormone functionality. *J Biol Chem* 288, 10230-10240
9. Jirácek, J., Záková, L., Antolíková, E., Watson, C. J., Turkenburg, J. P., Dodson, G. G., and Brzozowski, A. M. (2010) Implications for the active form of human insulin based on the structural convergence of highly active hormone analogues. *Proc Natl Acad Sci U S A* 107, 1966-1970
